# Boronate-Based Probes for Biological Oxidants: A Novel Class of Molecular Tools for Redox Biology

**DOI:** 10.3389/fchem.2020.580899

**Published:** 2020-09-25

**Authors:** Adam Sikora, Jacek Zielonka, Karolina Dębowska, Radosław Michalski, Renata Smulik-Izydorczyk, Jakub Pięta, Radosław Podsiadły, Angelika Artelska, Karolina Pierzchała, Balaraman Kalyanaraman

**Affiliations:** ^1^Faculty of Chemistry, Institute of Applied Radiation Chemistry, Lodz University of Technology, Lodz, Poland; ^2^Department of Biophysics and Free Radical Research Center, Medical College of Wisconsin, Milwaukee, WI, United States; ^3^Faculty of Chemistry, Institute of Polymer and Dye Technology, Lodz University of Technology, Lodz, Poland

**Keywords:** peroxynitrite, hydrogen peroxide, hydroperoxides, hypochlorous acid, azanone, NADPH oxidases, boronic acids, molecular probes

## Abstract

Boronate-based molecular probes are emerging as one of the most effective tools for detection and quantitation of peroxynitrite and hydroperoxides. This review discusses the chemical reactivity of boronate compounds in the context of their use for detection of biological oxidants, and presents examples of the practical use of those probes in selected chemical, enzymatic, and biological systems. The particular reactivity of boronates toward nucleophilic oxidants makes them a distinct class of probes for redox biology studies. We focus on the recent progress in the design and application of boronate-based probes in redox studies and perspectives for further developments.

## Introduction

In 1969, McCord and Fridovich discovered that erythrocuprein catalyzes the dismutation of the superoxide radical anion (${{\text{O}}_{2}}^{•-}$) (McCord and Fridovich, 1969), and the following year Sies and Chance (1970) showed that hydrogen peroxide is formed *in vivo*. Those discoveries attracted the attention of the scientific community to the role of reactive metabolites of molecular oxygen (so called reactive oxygen species, ROS) in physiological and pathophysiological processes. Over the last 50 years, tremendous progress has been made in understanding the biological chemistry of oxidants formed *in vivo*. Among them, the most important are hydrogen peroxide (H_2_O_2_) (Winterbourn, 2013), hypohalous acids (HOCl, HOBr) (Winterbourn, 2002; Rayner et al., 2014), hypothiocyanous acid (HOSCN) (Hawkins, 2009; Barrett and Hawkins, 2012), nitrogen dioxide (^•^NO_2_), carbonate radical anion (${{\text{CO}}_{3}}^{•-}$) (Augusto et al., 2002), and peroxynitrite (ONOO^−^) (Ferrer-Sueta et al., 2018). It is widely accepted that those oxidants are involved in different physiological and pathophysiological processes, but the detection and quantitation of those species remains a challenging task, mainly due to their high reactivity, short lifetime, and very low steady-state concentrations in biological systems. For that reason, fluorescent or chemiluminescent probes are widely used to study the role of biological oxidants in physiological and pathophysiological processes (Wardman, 2007; Winterbourn, 2014; Kalyanaraman et al., 2016, 2017; Hogg et al., 2017; Hardy et al., 2018). Several molecular probes that are oxidized to easily detectable and relatively stable fluorescent products have been developed, making possible real-time monitoring of the formation of biological oxidants. The latter is possible with the use of fluorescent and/or chromatographic techniques. Meaningful use of those probes, and proper interpretation of experimental data obtained, requires a good understanding of the mechanism of their action. One of the most important pre-requisites is determination of the probe reactivity pattern.

**Scheme 1:**
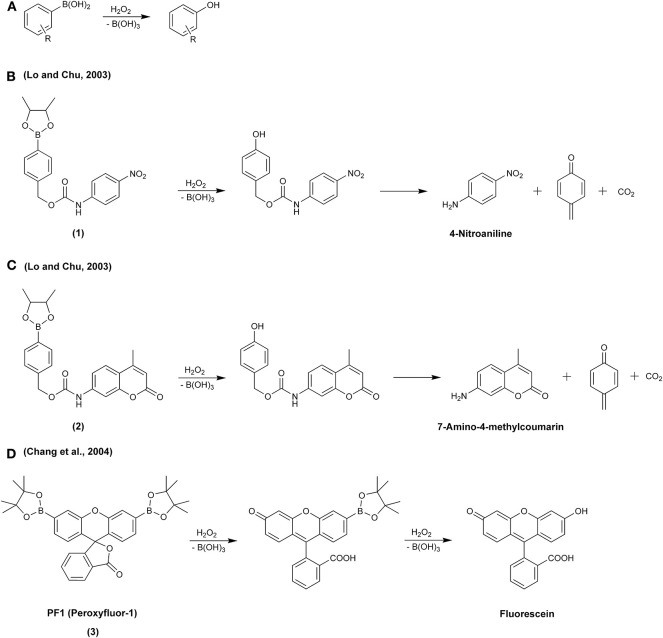
**(A)** Oxidation of aryl boronates to phenolic products in the reaction with H_2_O_2_. **(B–D)** Reaction of H_2_O_2_ with molecular boronate probes: **(B)** *p*-dihydroxyborylbenzyloxycarbonyl derivative of 4-nitroaniline, **(C)** *p*-dihydroxyborylbenzyloxycarbonyl derivative of 7-amino-4-methylcoumarin, **(D)** diboronate derivative of fluorescein, PF1.

In the middle of the first decade of the 21st century, a new class of fluorogenic probes, designed for the detection of H_2_O_2_ and based on the oxidation of boronate derivatives of fluorescent dyes, was developed (Lo and Chu, 2003; Chang et al., 2004). The design was based on the previously reported oxidative deboronation of aryl boronates to the corresponding phenolic products (Scheme 1A) (Ainley and Challenger, 1930; Kuivila, 1954). Since then, boronate-based molecular probes have emerged as potential tools for detecting several biological oxidants: H_2_O_2_ (Lo and Chu, 2003; Chang et al., 2004), peroxynitrite (Sikora et al., 2009), hypochlorous acid (Sikora et al., 2009), organic hydroperoxides (Michalski et al., 2014), and peroxymonocarbonate (Truzzi and Augusto, 2017; Rios et al., 2020). This relatively simple oxidation reaction has been utilized in the design of a wide variety of redox probes, with the detection modalities including spectrophotometry (Lo and Chu, 2003; Zhan et al., 2010; Lu et al., 2011; Choudhury et al., 2019), fluorescence (Chang et al., 2004; Miller et al., 2005, 2007; Dickinson et al., 2010; Zielonka et al., 2012b), chemiluminescence (Green et al., 2017a,b; Seven et al., 2017; Gnaim and Shabat, 2019; Calabria et al., 2020), bioluminescence (Van De Bittner et al., 2010; Wu et al., 2014; Szala et al., 2020), high-performance liquid chromatography (HPLC) (Sikora et al., 2009, 2013; Zielonka et al., 2014), mass spectrometry (Cochemé et al., 2011; Zielonka et al., 2016b), and PET (positron emission tomography) imaging (Carroll et al., 2014). Also, theranostic (therapeutic + diagnostic) strategies to release a bioactive agent in combination with a reporting moiety (e.g., fluorescent, chemiluminescent) using a boronate group as an oxidant-sensitive trigger have been proposed (Biswas et al., 2017; Gnaim and Shabat, 2019; Odyniec et al., 2019).

The history of the development of boronate-based probes for their use in redox biology began in 2003, when Lo and Chu (2003) proposed that *p-*dihydroxyborylbenzyloxycarbonyl derivatives of *p-*nitroaniline (**1**) and fluorescent 7-amino-4-methylcoumarin (**2**) could be used as probes for the selective detection of H_2_O_2_. The *p-*dihydroxyborylbenzyloxycarbonyl group, an amine protecting group that can be selectively removed by H_2_O_2_, has been known in organic chemistry since 1975 (Kemp and Roberts, 1975). The proposed mechanism of H_2_O_2_ detection was based on the oxidative deprotection of *p-*nitroaniline (λ_max_ = 383 nm, Scheme 1B) or fluorescent 7-amino-4-methylcoumarin (λ_ex_ = 348 nm, λ_em_ = 440 nm, Scheme 1C). In 2004, the synthesis and characterization of a diboronate derivative of fluorescein, PF1 (**3**), was described (Scheme 1D) (Chang et al., 2004). The probe was shown to undergo oxidation in live cells exposed to exogenously added H_2_O_2_, with the formation of a green fluorescent product detectable using fluorescence microscopy.

**Figure 1:**
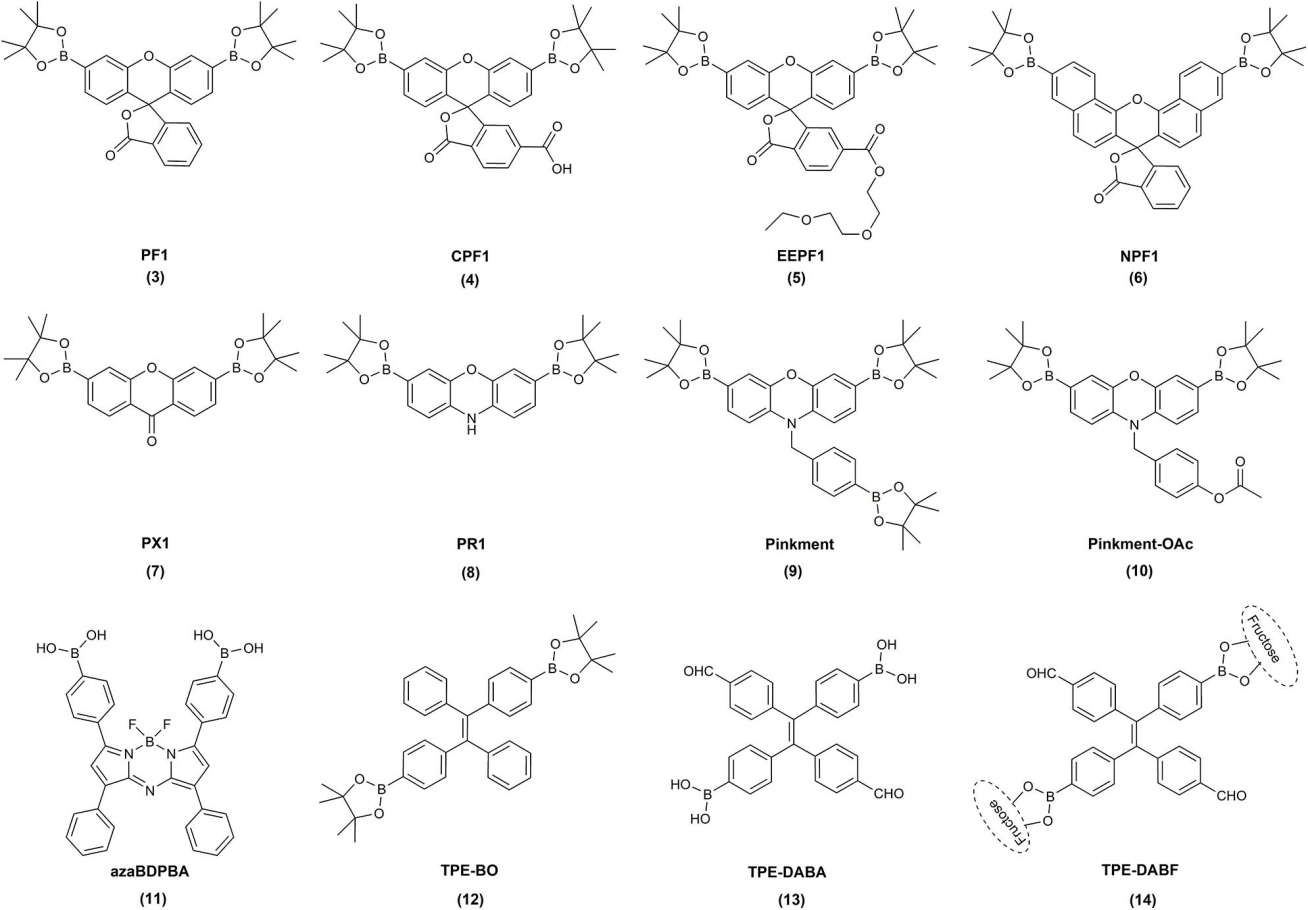
Examples of the chemical structures of the reported diboronate fluorogenic probes for biological oxidants.

**Figure 2:**
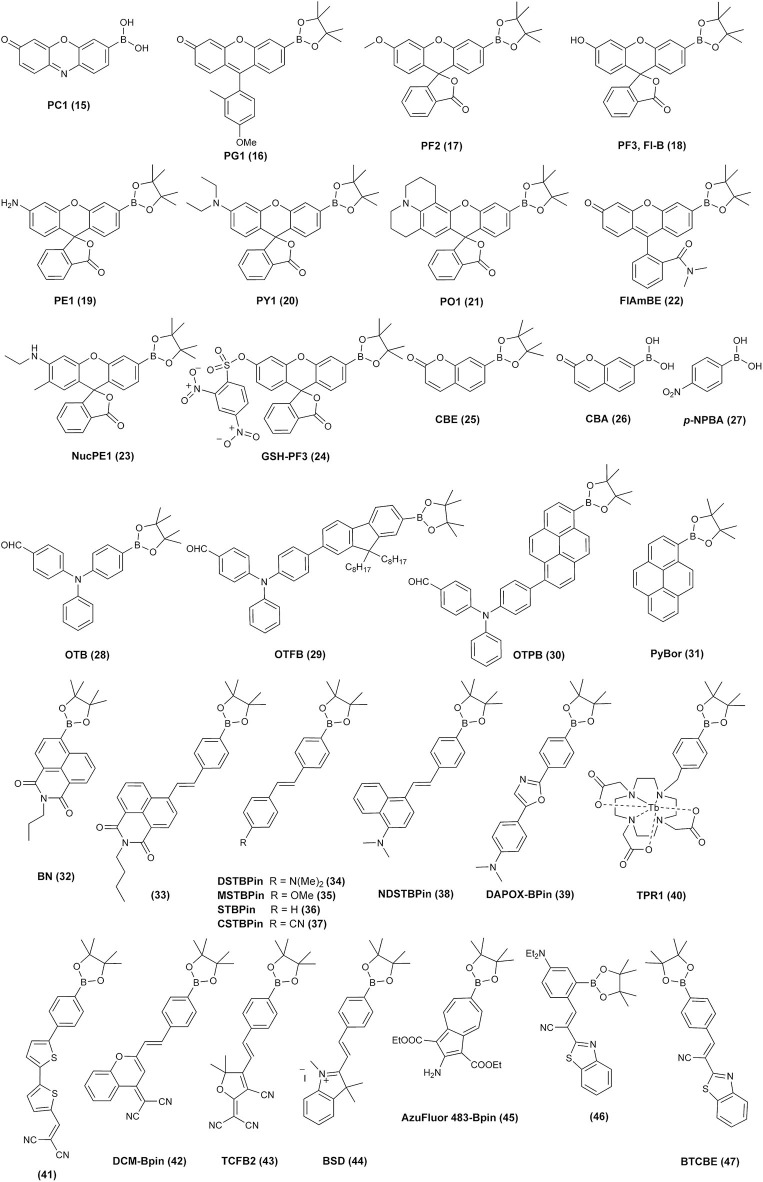
Examples of the chemical structures of the reported monoboronate fluorogenic or colorimetric probes for biological oxidants.

Following these reports, over the last 15 years, a great variety of boronate-based molecular probes designed for the detection of H_2_O_2_, hypochlorite, or peroxynitrite were described in the literature. The Chang laboratory pioneered the development of boronate-based fluorogenic probes. In an elegant series of studies, the Chang lab described the design, synthesis, and characterization of several diboronate fluorogenic probes: fluorescein-based PF1 (Chang et al., 2004), resorufin-based PR1 (**8**) (Miller et al., 2005), xanthone-based PX1 (**7**) (Miller et al., 2005), and naphthofluorescein-based NPF1 (**6**) (Albers et al., 2008) (Figure 1). Introduction of two boronate groups, due to the synthetic constrains, however, limited the sensitivity of the probes in biological settings, as two consecutive oxidations were required to unmask the fluorophore (Scheme 1D). Therefore, the same group subsequently developed and reported the synthesis and characterization of a broad palette of monoboronate fluorogenic probes with varying emission colors: Tokyo Green-based PG1 (**16**) (Miller et al., 2007); resorufin-based PC1 (**15**) (Miller et al., 2007); and fluorescein- or rhodol-based PF2 (**17**), PF3 (**18**), PE1 (**19**), PY1 (**20**), and PO1 (**21**) (Dickinson et al., 2010) (Figure 2). This class of probes enabled monitoring of endogenously produced cellular oxidants (Miller et al., 2007).

**Scheme 2:**
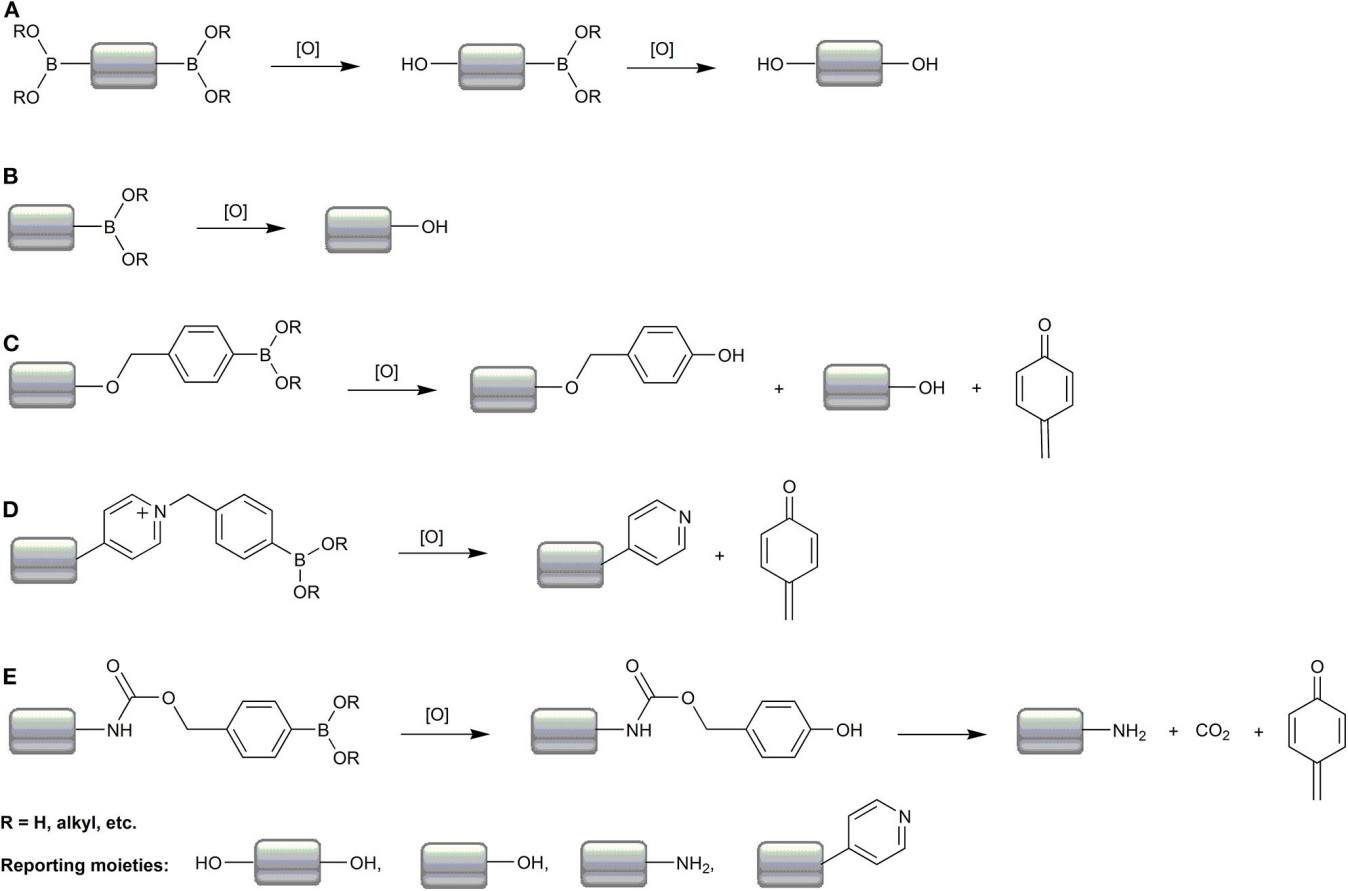
Summary of the mechanisms of oxidative deboronation of boronate-based molecular probes. Most probes are based on direct formation of the phenolic reporters from diboronate **(A)** and monoboronate **(B)** probes or on self-immolation chemistry upon oxidation of the boronobenzyl **(C,D)** or boronobenzyloxycarbonyl **(E)** derivatives of the reporting moieties.

**Figure 3:**
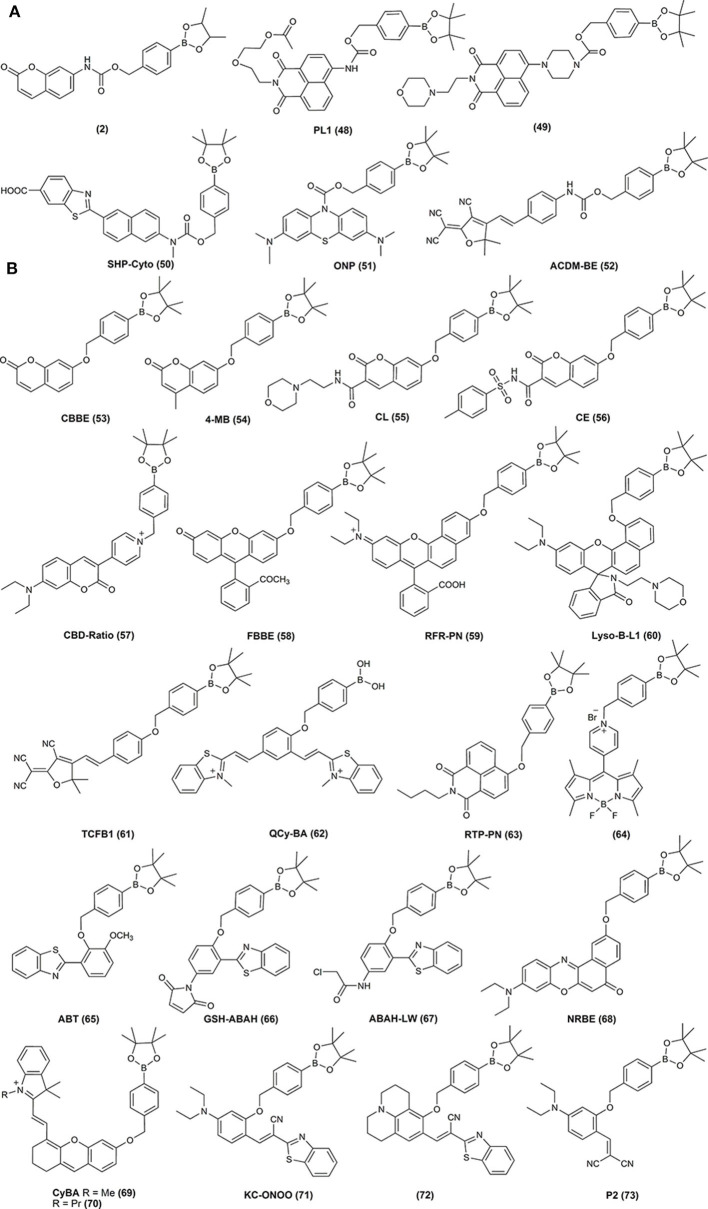
Examples of the chemical structures of boronobenzyloxycarbonyl **(A)** and boronobenzyl **(B)** derivatives of fluorescent dyes reported as probes for biological oxidants.

While the chemistry of the oxidant sensing event is the same for most boronates, as shown in Scheme 1A, and involves oxidative deboronation that leads to the formation of the reporting molecule, at least four classes of boronate-based molecular probes can be distinguished based on their structure and the mechanism of the formation of the detectable product:

1. Diboronate probes, where the formation of the fluorescent reporting molecule requires oxidation of two boronate groups (Scheme 2A). Several examples of this class of probes are shown in Figure 1, and the spectroscopic properties of the detectable products are summarized in Table 1. The main disadvantage of these probes is their low sensitivity due to the requirement of two consecutive oxidation reactions.
2. Monoboronate probes, where the direct and stoichiometric (1:1) oxidation of the probe by biological oxidant leads to the formation of the detectable (in most cases fluorescent) reporter (Scheme 2B). The examples of this class of probes are shown in Figure 2, and the spectroscopic properties of the oxidation products are summarized in Table 1.
3. Boronobenzyl probes, where oxidation of the probe leads to the formation of 4-hydroxybenzyl derivative of the reporting molecule. The primary phenolic product undergoes subsequent spontaneous release of the fluorescent product *via* elimination of the quinone methide (QM) moiety (Schemes 2C,D). Examples of this class of probes are shown in Figure 3, and the spectroscopic properties of the end products are summarized in Table 2. The most important advantage of these probes is a straightforward one-step synthetic procedure using commercial materials, which enables relatively easy probe preparation.
4. *p-*Dihydroxyborylbenzyloxycarbonyl probes, where oxidation of the probe leads to the formation of the primary phenolic product, which upon subsequent elimination of QM and carbon dioxide (CO_2_) unmasks the amine group in the reporting molecule. This typically results in an increase (“turn-on”) in the fluorescence yield (Scheme 2E). Examples of this class of probes are shown in Figure 3, and the spectroscopic properties of the products formed are summarized in Table 2.

**Table 1:** Spectroscopic properties of diboronate and monoboronate molecular probes for detection of biological oxidants.

**No**.	**Name**	**Probe**			**Oxidation product**			**Comment**	**References**
		**λ_abs_ (nm)**	**ε (M^**−1**^cm^**−1**^)**	**λ_em_ (nm); ϕ**	**λ_exc_ (nm)**	**ε (M^**−1**^cm^**−1**^)**	**λ_em_ (nm); ϕ**		
**DIBORONATE PROBES**									
**3**	PF1	NA	-	NA	450^a^	-	ca. 515^a^		Chang et al., 2004
**4**	CPF1	-	-	-	450	-	520		Purdey et al., 2015
**5**	EEPF1	-	-	-	450	-	520		Purdey et al., 2015
**6**	NPF1	345^i^	22 400^i^	-	598^i^	-	660^i^		Albers et al., 2008
**7**	PX1	350^a^	4 700^a^	400(weak)^a^	350^a^	-	ca. 450^a^		Miller et al., 2005
**8**	PR1	NA	-	NA	530^a^	-	ca. 580^a^		Miller et al., 2005 Weber et al., 2018
**9**	Pinkment	-	-	-	545^b^	-	ca. 580		Odyniec et al., 2018
**10**	Pinkment-OAC	-	-	-	545^b^	-	ca. 580		Odyniec et al., 2018
**11**	azaBDPBA	655	-	682; 0.6	-	-	724^j^		Liu et al., 2015
**12**	TPE-BO	-	-	-	ca. 400	-	ca. 500	AIE-fluorescence	Zhang et al., 2015
**13**	TPE-DABA	-	-	-	380^b^		ca. 500^b^	AIE-fluorescence	Liu et al., 2016
**14**	TPE-DABF	ca. 270 ca. 350	-	-	430^b^		576^b^	AIE-fluorescence	Liu et al., 2016
**MONOBORONATE PROBES**									
**15**	PC1	480^a^	4800^a^	584^a^; 0.006^a^	550^a^	-	-	*k*(H_2_O_2_) ≈ 1 M^−1^s^−1a^ *k*(HOCl) = 1.1 × 10^4^ M^−1^s^−1b^ *k*(ONOO^−^) = 1 × 10^6^ M^−1^s^−1b^	Miller et al., 2007 Debowska et al., 2016
**16**	PG1	460^a^	5500^a^	510^a^; 0.075^a^	450^a^	-	-	*k*(H_2_O_2_) ≈ 1.1 M^−1^s^−1a^	Miller et al., 2007
**17**	PF2	n/a	n/a	n/a	475^a^	28 600^a^	511; 0.27^a^	*k*(H_2_O_2_) ≈ 0.47 M^−1^s^−1a^	Dickinson et al., 2010
**18**	PF3, Fl-B	454^a^	24 000^a^	521; 0.1^a^	492^a^	88 000^a^	515; 0.94^a^	*k*(H_2_O_2_) ≈ 0.38 M^−1^s^−1a^ *k*(H_2_O_2_) = 0.65 M^−1^s^−1b^ *k*(HOCl) = 4.3 × 10^2^ M^−1^s^−1b^ *k*(ONOO^−^) = 1.1 × 10^6^ M^−1^s^−1b^	Dickinson et al., 2010 Rios et al., 2016
**19**	PE1	480^a^	16 400^a^	519; 0.3^a^	491^a^	51 200^a^	514; 0.93^a^	*k*(H_2_O_2_) ≈ 0.81 M^−1^s^−1a^	Dickinson et al., 2010
**20**	PY1	494^a^	16 200^a^	558; 0.1^a^	519^a^	48 900^a^	548; 0.12^a^	*k*(H_2_O_2_) ≈ 0.37 M^−1^s^−1a^	Dickinson et al., 2010
**21**	PO1	507^a^	13 900^a^	574; 0.07^a^	540^a^	29 300^a^	565; 0.46^a^	*k*(H_2_O_2_) ≈ 0.52 M^−1^s^−1a^	Dickinson et al., 2010
**22**	FlAmBE	-	-	-	485^b^	-	535^b^	*k*(ONOO^−^) > 5 × 10^5^ M^−1^s^−1^	Zielonka et al., 2012b Zielonka et al., 2012a
**23**	NucPE1	468^a^ 490^a^	27 300^a^ 26 000^a^	530; 0.117^a^	505^a^	19 100^a^	530; 0.626^a^	*k*(H_2_O_2_) ≈ 0.82 M^−1^s^−1a^	Dickinson et al., 2011b
**24**	GSH-PF3	-	-	-	488	-	512	ONOO^−^ and GSH detection	Sedgwick et al., 2018
**25**	CBE	-	-	-	332	-	450	-	Du et al., 2008
**26**	CBA	287^b^	12 000^b^	-	324^b^	13 000^b^	450^b^	*k*(H_2_O_2_) = 1.5 M^−1^s^−1b^; *k*(ONOO^−^) = 1.1 × 10^6^ M^−1^s^−1b^	Zielonka et al., 2010 Zielonka et al., 2012b
**27**	*p*-NPBA	294^c^	-	-	405^c^	19 400^c^	-	Colorimetric probe	Lu et al., 2011
**28**	OTB	-	-	-	366	-	466	The organic thin-film fluorescence probe for sensing of hydrogen peroxide vapor	Fu et al., 2016
**29**	OTFB	-	-	-	-	-	-	The organic thin-film fluorescence probe for sensing of hydrogen peroxide vapor	Fu et al., 2016
**30**	OTPB	-	-	-	-	-	-	The organic thin-film fluorescence probe for sensing of hydrogen peroxide vapor	Fu et al., 2016
**31**	PyBor	265^b^ 275^b^ 325^b^ 340^b^	41 550^b^ 69 970^b^ 44 120^b^ 59 450^b^	377, 392, 417; 0.08^b^	268^b^ 280^b^ 350^b^ 360^b^ 385^b^	55 160^b^ 95 610^b^ 65 280^b^ 29 350^b^ 25 190^b^	396, 410, 430; 0.60^b^		Yu et al., 2012
**32**	BN	344^d^	-	455^d^	455^d^	-	555^d^	Probe designed for ACh detection	Liu et al., 2014
**33**	-	376^e^	-	536; 0.54^e^	424^e^	-	604, 0.18^e^	Ratiometric probe	Lee et al., 2019
**34**	DSTBPin	ca. 355^f^	-	488^f^	350^f^	-	444^f^	Increase in fl. intensity	Lampard et al., 2018
**35**	MSTBPin	ca. 330^f^	-	405^f^	330^f^	-	380^f^	Increase in fl. intensity	Lampard et al., 2018
**36**	STBPin	ca. 320^f^	-	ca.360^f^	315^f^	-	ca. 360^f^	Small decrease in fl. Intensity	Lampard et al., 2018
**37**	CSTBPin	ca. 330^f^	-	ca. 380^f^	330^f^	-	ca. 380^f^	Decrease in fl. Intensity	Lampard et al., 2018
**38**	NDSTBPin	360^f^	-	525^f^	350^f^	-	ca. 510^f^	Decrease in fl. Intensity	Lampard et al., 2018
**39**	DAPOX-Bpin	350^f^	-	480^f^	350^f^	-	475^f^	Increase in fl. intensity	Lampard et al., 2018
**40**	TPR1	226^b^	6700^b^	545; 0.029^b^	280^b^	-	545; 0.054^b^	Terbium-based fluorogenic probe	Lippert et al., 2010
**41**		463	36 500	565	482	-	640	Ratiometric I_640_/I_565_; probe designed for HOCl detection	Tong et al., 2018
**42**	DCM-Bpin	434	-	-	560	-	667		Wu L. et al., 2019
**43**	TCF-OCl, TCFB2	400	-	590	560	-	606	Probe designed for HOCl detection	Shu et al., 2015 Sedgwick et al., 2017 Choudhury et al., 2019
**44**	BSD	391^g^	-	-	522^g^	87 000^g^	-	Colorimetric probe	Zhan et al., 2010
**45**	AzuFluor 483-Bpin	327^f^	-	-	335^f^	-	483		Murfin et al., 2019
**46**		478	61 300	-	ca. 480	>61 300	522	*k_*obs*_*(ONOO^−^) = 4.2 × 10^3^ M^−1^s^−1^ Formation of the fluorophore *via* intramolecular cyclization of oxidation product	Zhang J. et al., 2016
**47**	BTCBE	356^h^	-	-	441^h^	-	522; 0.088^h^		Shu et al., 2020a

a *20 mM HEPES buffer, pH 7, 25°C*.

b *Phosphate buffer, pH 7.4, 25°C*.

c *75 mM carbonate/bicarbonate buffer, pH 9.0*.

d *Phosphate buffer, pH 7.5*.

e *10 mM phosphate buffer, pH 7.4, containing 50% (v/v) DMSO*.

f *H_2_O/CH_3_OH (52.1 wt%), pH 8.2*.

g *NaHCO_3_/Na_2_CO_3_ buffer, pH 9*.

h *PBS/ethanol = 7:3, pH 7.4*.

i *20 mM HEPES buffer, pH 7.5*.

j *PBS/ethanol 1:1, pH 7.4*.

**Table 2:** Spectroscopic properties of boronobenzyl derivatives of fluorescent dyes reported as probes for biological oxidants.

**No**.	**Name**	**Probe**			**Oxidation product**			**Comment**	**References**
		**λ_abs_ (nm)**	**ε (M^**−1**^cm^**−1**^)**	**λ_em_ (nm); ϕ**	**λ_exc_ (nm)**	**ε (M^**−1**^cm^**−1**^)**	**λ_em_ (nm); ϕ**		
**2**		348^a^		440^a^	355^a^		460^a^		Lo and Chu, 2003
**48**	PL1	375^b^	9600^b^	475; 0.38^b^	435^b^	8600^b^	540; 0.11^b^	*k*(H_2_O_2_) ≈ 0.88 M^−1^s^−1^; ratiometric probe	Srikun et al., 2008
**49**		400^c^		528^c^	400^c^		528^c^	Lysosome targetable probe; increase in fl. Intensity	Kim et al., 2015
**50**	SHP-Cyto	333^d^	38,000^d^	455; 1.00^d^	370^d^	21,000^d^	528; 0.7^d^	Ratiometric probe; two-photon also	Lim et al., 2018
**51**	ONP	640^e^	29,160^e^	692; 0.007^e^	665^e^		692; 0.11^e^	Increase in fl. Intensity	Hu et al., 2019
**52**	ACDM-BE	ca. 445^f^			540^f^		604^f^		Wang G. et al., 2020
**53**	CBBE	ca.330^g^		Non-fluorescent	370^g^		453^g^	*k*(H_2_O_2_) ≈ 0.46 M^−1^s^−1^	Daniel et al., 2013
**54**	4-MB	320^h^		385^h^	370^h^		450; 0.31^h^		Palanisamy et al., 2017
**55**	CL			395^i^	340^i^		447^i^	Ratiometric probe	Weber et al., 2020
**56**	CE			405^i^	340^i^		447^i^	Ratiometric probe	Weber et al., 2020
**57**	CBD-Ratio	497^j^		565^j^	450^j^		500^j^	Ratiometric probe	Li et al., 2019
**58**	FBBE	456^k^ 483^k^	25,000^k^ 22,000^k^	Non-fluorescent	494^k^	78,000^k^	518^k^	*k*(ONOO^−^) = 2.8 × 10^5^ M^−1^s^−1^ *k*(H_2_O_2_) = 0.96 M^−1^s^−1^ *k*(OCl^−^) = 8.6 × 10^3^ M^−1^s^−1^	Debowska et al., 2016
**59**	RFR-PN	ca.577^i^		560^i^	480^i^		630^i^	Ratiometric probe	Zhu et al., 2018
**60**	Lyso-B-L1			Non-fluorescent	570^l^		606^l^		Liu et al., 2017
**61**	TCFB1	ca.440^m^			560^m^		606; 0.002^m^		Sedgwick et al., 2017
**62**	QCy-BA	400^i^		565^i^	ca. 480 400 (with DNA)^i^		ca.680 650(with DNA)^i^	Binding to DNA	Narayanaswamy et al., 2016
**63**	RTP-PN	372^i^		450^i^	415^i^		543^i^	Ratiometric probe	Wang Z. et al., 2019
**64**		508^i^		520; 0.002^i^	480^i^		520; 0.78^i^		Xu et al., 2014
**65**	ABT	309^n^		405^n^	317^n^		483^n^	*k*(ONOO^−^) = 1.16 × 10^4^ M^−1^s^−1^ *k*(H_2_O_2_) = 0.23 M^−1^s^−1^ *k*(OCl^−^) = 4.81 M^−1^s^−1^	Li and Yang, 2018
**66**	GSH-ABAH	326^o^		Non-fluorescent	390^o^		451^o^	GSH and ONOO detection	Wu et al., 2018a
**67**	ABAH-LW	ca. 430,490^o^		405^o^	370^o^		481^o^	Ratiometric probe; increase in fl. Intensity	Wu et al., 2018b
**68**	NRBE			670; 0.01^p^	585^p^		670; 0.36^p^		Diao et al., 2019
**69**	CyBA	610, 660			700			Photoacoustic imaging	Hariri et al., 2019
**70**		ca. 610, 650, 740^q^		708^q^	670^q^		708^q^	Increase in fl. Intensity	Zhang et al., 2019
**71**	KC-ONOO	510^r^	33,000^r^	<0.01^r^	480	34,000^r^	530; 0.24^r^		Xia et al., 2019
**72**		468^s^	20,370^s^	646; 0.01^s^	500^s^	29,500^s^	540; 0.62^s^	*k*(ONOO^−^) ≈ 1.51 × 10^4^ M^−1^min^−1^ *k*(H_2_O_2_) ≈ 2.24 M^−1^min^−1^	Kim J. et al., 2014
**73**	P2	440^t^		580^t^	440^t^		480^t^	*k*(ONOO^−^) = 7.65 × 10^3^ M^−1^min^−1^ *k*(H_2_O_2_) = 75.3 M^−1^min^−1^	Zhou et al., 2014

a *140 mM NaHCO_3_, pH 8.3, 25°C*.

b *20 mM HEPES, pH 7.4*.

c *0.1 M PBS, pH 7.4, 1% DMF*.

d *30 mM MOPS buffer, 100 mM KCl, pH 7.4*.

e *10 mM PBS, 5% CH_3_CN, pH 7.4, 37°C*.

f *CH_3_CN/PBS (0.1 M, pH 7.4, v/v, 3/20)*.

g *50 mM HEPES buffer, pH 7.5*.

h *20 mM HEPES, 1% DMSO, pH 7.4 25°C*.

i *PBS, pH 7.4, 25°C*.

j *20% DMSO, 20 mM PBS, pH 7.4*.

k *50 mM phosphate buffer, pH 7.4, 100 μM dtpa, 10% CH_3_CN*.

l *10 mM acetate buffer, 1% DMSO, pH 4.5*.

m *PBS, 20% DMSO, pH 8.0*.

n *10 mM PBS, pH 7.4, 40% EtOH*.

o *PBS, pH 8.2, 8% DMSO, 1 mM CTAB*.

p *HEPES buffer 0.1 M, pH 7.4*.

q *PBS, pH 7.4, 37°C*.

r *10 mM PBS, 10% DMSO, pH 7.4*.

s *10 mM phosphate buffer, pH 7.4, 10% C_2_H_5_OH, 37°C*.

t *10 mM PBS, 10% CH_3_CN, pH 7.4*.

## Synthetic Approaches to Boronate-Based Molecular Probes

**Scheme 3:**
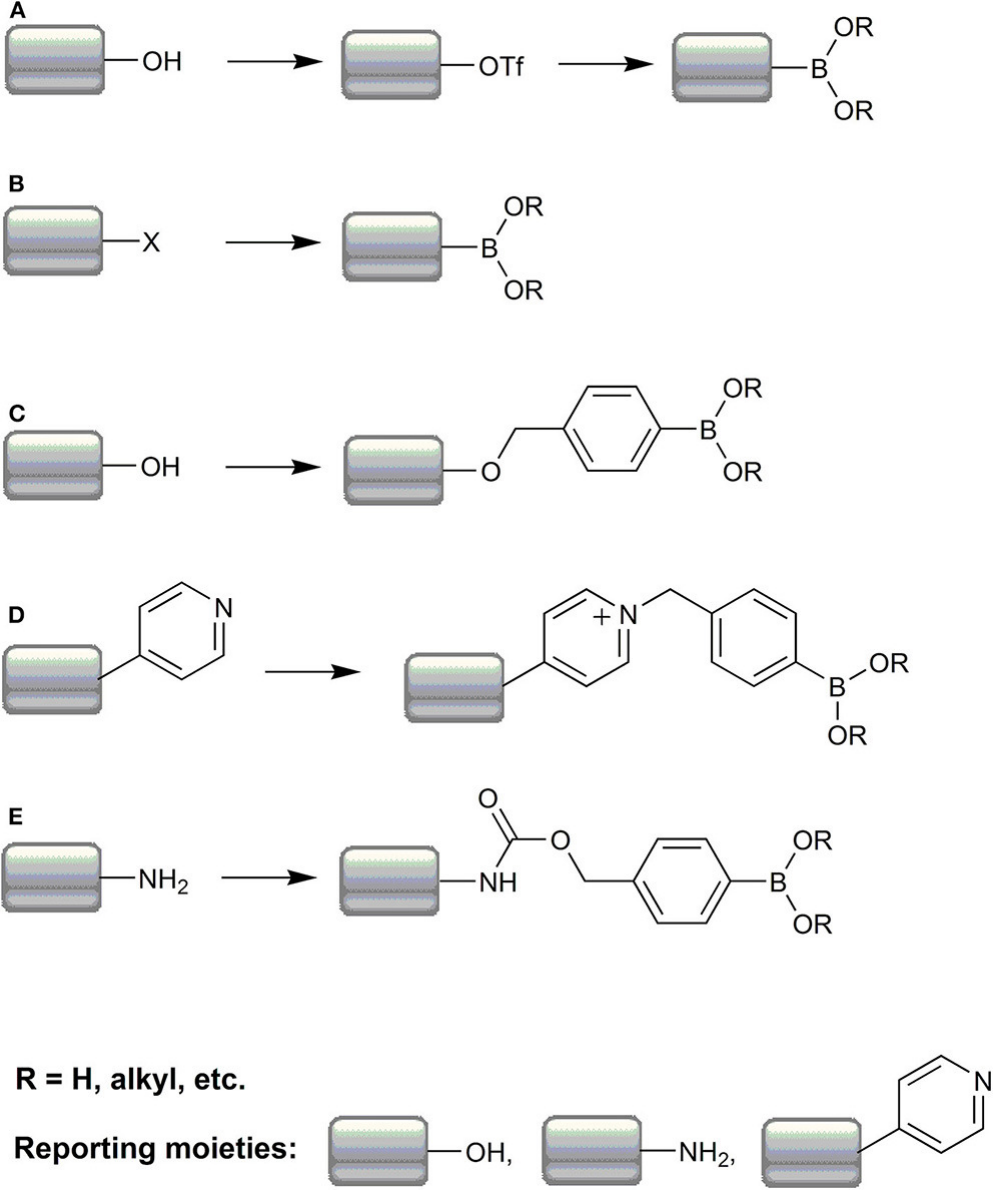
Summary of the most frequent strategies in the synthesis of boronate-based redox probes. The typical pathways of the probe synthesis include conversion of the phenolic hydroxyl group to the boronate moiety via a triflate intermediate **(A)**, conversion of aryl halides to arylboronates **(B)**; boronobenzylation of phenols **(C)** or heterocyclic nitrogen **(D)**, and boronobenzyloxycarbonylation of aromatic and aliphatic amines **(E)**.

In the case of boronate-based fluorogenic probes, the introduction of a boronic acid or ester group directly or by introduction of the boronobenzyl or *p-*dihydroxyborylbenzyloxycarbonyl moieties, can be considered as fluorescence masking or a fluorescence “on/off” molecular switch. Synthetic protocols for boronate-based probes can be roughly divided into five strategies: (i) substitution of an aromatic hydroxyl group *via* triflate intermediate (Scheme 3A), (ii) substitution of an aromatic halogen atom (Scheme 3B), (iii) “boronobenzylation” of a phenolic compound (Scheme 3C) or heterocyclic nitrogen atom (represented as a pyridine nitrogen) (Scheme 3D), (iv) synthesis of a *p-*dihydroxyborylbenzyloxycarbonyl derivative of aromatic amine (Scheme 3E), and (v) miscellaneous syntheses.

### Substitution of a Hydroxyl Group in an Aromatic Ring

In this strategy, the boronic moiety is introduced indirectly *via* transformation of an aromatic hydroxyl group (phenol) into a good leaving group in a reaction with trifluoromethanesulfonic anhydride (Demicheli et al., 2016; Zhang J. et al., 2016) or *N*-phenyl-bis(trifluoromethanesulfonimide) (Albers et al., 2008; Srikun et al., 2010; Kim E. J. et al., 2014) in the presence of amines like DMAP (4-dimethylaminopyridine) (Demicheli et al., 2016), DIPEA (N,N-diisopropylethylamine) (Rios et al., 2016), or pyridine (Li et al., 2020) in anhydrous solvent under inert atmosphere at room temperature. Subsequently, the triflate derivative is substituted in a Pd(dppf)Cl_2_-assisted reaction with bis(pinacolato)diboron in the presence of potassium acetate, and refluxed under anaerobic conditions (Scheme 3A) (Miller et al., 2007; Albers et al., 2008; Srikun et al., 2010).

### Substitution of a Halogen Atom in the Aromatic Ring

This approach is more direct and, in the majority of cases, can be described as a straightforward bromide (Miller et al., 2005; Albers et al., 2006; Mohapatra and Phillips, 2012), rarely iodide (Chang et al., 2004; McQuaker et al., 2013), or chloride (Zhu B. et al., 2013), substitution in a reaction with bis(pinacolato)diboron in the presence of potassium acetate and Pd(dppf)Cl_2_ in various solvents at elevated temperature and under inert atmosphere (Scheme 3B). It is noteworthy that, in some rare cases, a strategy in which aryl halides undergo reaction with *n*-BuLi in −78°C followed by reaction with alkylborates [e.g., trimethylborate (Lo and Chu, 2003), triisopropylborate (Yu et al., 2012), isopropylpinacolborate (Quin et al., 2010; McQuaker et al., 2013)] can be used to obtain corresponding boronic acid derivatives.

### Boronobenzylation of Phenols or Heterocyclic Nitrogen

The most broadly used reaction in syntheses of the desired boronic probes consists of the so-called boronobenzylation, in which a phenol residue reacts, usually in an inert atmosphere, with 2-(4-bromomethylphenyl)-4,4,5,5-tetramethyl-[1,3,2]dioxaborolane (also known as 4-bromomethylphenylboronic acid pinacol ester) in the presence of a base, typically K_2_CO_3_ (Karton-Lifshin et al., 2012; Kim J. et al., 2014; Zhou et al., 2014) or Cs_2_CO_3_ (Van De Bittner et al., 2010; Qian et al., 2012; Yuan et al., 2012), in an anhydrous medium (Scheme 3C). There are also few literature examples of a 4-iodo-derivative usage (Karton-Lifshin et al., 2012; Debowska et al., 2016; Narayanaswamy et al., 2016). A variety of solvents can be used and usually the reaction is performed under reflux during a couple of hours or is left at room temperature until completion.

There are also examples of a probe synthesis in which the boronobenzyl moiety is introduced directly to an aromatic ring nitrogen in a pyridine (Xu et al., 2014; Li et al., 2019) or quinoline (He et al., 2018) system as well as onto aliphatic amine nitrogen (Scheme 3D) (Sun et al., 2014; Li K.-B. et al., 2017; Han et al., 2018). However, in the rearmost examples, 2-bromomethyl-derivative is used.

### Synthesis of Carbamate Type Boronic Probes

The fluorescence emitting form of some probes consists of an aniline-type, exocyclic nitrogen atom. In such cases, the amine group is often masked with a carbamic acid 4-(4,4,5,5-tetramethyl-[1,3,2]dioxaborolan-2-yl)-benzyl ester moiety (Scheme 3D), decreasing the electron density on the nitrogen atom. The typical procedure to introduce such a residue exploits the 4-hydroxymethylphenylboronic acid pinacol ester in which the hydroxyl group is converted, often *in situ*, into an acid chloride in a reaction with triphosgene (Srikun et al., 2008; Chung et al., 2011; Zhu D. et al., 2013) in the presence of a base in various solvents, which reacts further with the target amino group. There are some examples in which this approach is also used to mask the nitrogen atom on biotin (Chung et al., 2018; Wu Y. P. et al., 2018) or methylene blue (Hu et al., 2019) or in aliphatic systems (Lippert et al., 2010; Zhu D. et al., 2013; Yik-Sham Chung et al., 2018).

### Miscellaneous Syntheses

In this review, we would be remiss to omit some examples of formylphenylboronic ester/acid usage in carbon-carbon bond formation. In those reactions, which are performed mostly in boiling anhydrous ethanol (Wang et al., 2013; Guo et al., 2018), the oxygen atom of the aldehyde group serves as a base (in some cases, DIPEA (Shu et al., 2020a,b) or piperidine (Wu L. et al., 2019) is added), which abstracts an acidic proton from the reaction partner and the negatively charged carbon atom obtained attacks the protonated carbonyl group with subsequent double bond formation. For example, a single bond formation serves the reductive amination reaction in which a 2-(2-aminophenyl)benzothiazole reacts with the 4-formylphenylboronic pinacol ester in the presence of NaBH(OAc)_3_ (Sedgwick et al., 2016).

Also, the Wittig reaction is applied in double bond formation (Das et al., 2009; Lee et al., 2019). In this approach, the 4-bromophenylboronic pinacol ester is transformed into an ylide in a direct reaction with triphenylphosphine, carried out in reflux under anaerobic conditions in the dark, which is used in the subsequent reaction with an aldehyde group of the reporter residue.

## Subcellular Targeting of Boronate Probes

Fluorescence microscopy enables spatial resolution at the level of cellular organelles, and many boronate probes have been designed to accumulate in a specific organelle for site-specific detection of H_2_O_2_. Typically, an organelle-targeting moiety is covalently attached to a probe for that purpose (Xu W. et al., 2016; Zhu et al., 2016; Gao et al., 2019). Examples of the cellular organelles that have been targeted to accumulate boronate-based redox probes include mitochondria (see below), nucleus (Dickinson et al., 2011b; Wen et al., 2014), endoplasmic reticulum, Golgi apparatus (Wu et al., 2018b; Wang H. et al., 2019), and lysosomes (Kim et al., 2015; Liu et al., 2017; Ren et al., 2017). Also, simultaneous monitoring of H_2_O_2_ in mitochondria and endoplasmic reticulum has been reported using a combination of targeted probes, yielding products of different fluorescence parameters (Xiao et al., 2016). In addition to targeting a specific organelle, boronate redox probes designed for intracellular accumulation and retention have been developed, with the aim of differentiating between intracellular and extracellular oxidants (Miller et al., 2010; Dickinson et al., 2011a).

By far, the highest number of targeted boronate redox probes was designed and reported for the detection of mitochondrial oxidants. Detection and quantitation of biological oxidants produced inside mitochondria remain challenging, and numerous mitochondria-targeted redox probes were designed and synthesized for that purpose (Zielonka et al., 2017b; Cheng et al., 2018), among them a series of boronate-based molecular probes. Mitochondria are regarded as one of the major cellular sources of ${{\text{O}}_{2}}^{•-}$, which undergoes spontaneous or superoxide dismutase-catalyzed dismutation to H_2_O_2_. It is also possible that ${{\text{O}}_{2}}^{•-}$ reacts in mitochondria with nitric oxide (^•^NO), as ^•^NO can easily diffuse across lipid membranes, to produce peroxynitrite—a significantly faster oxidant of the boronate probes. The ability to directly and stoichiometrically react with H_2_O_2_ or ONOO^−^, without the requirement of a catalyst is a significant advantage of the boronate-based probes, as compared with many other probes used for the detection of mitochondrial oxidants.

**Figure 4:**
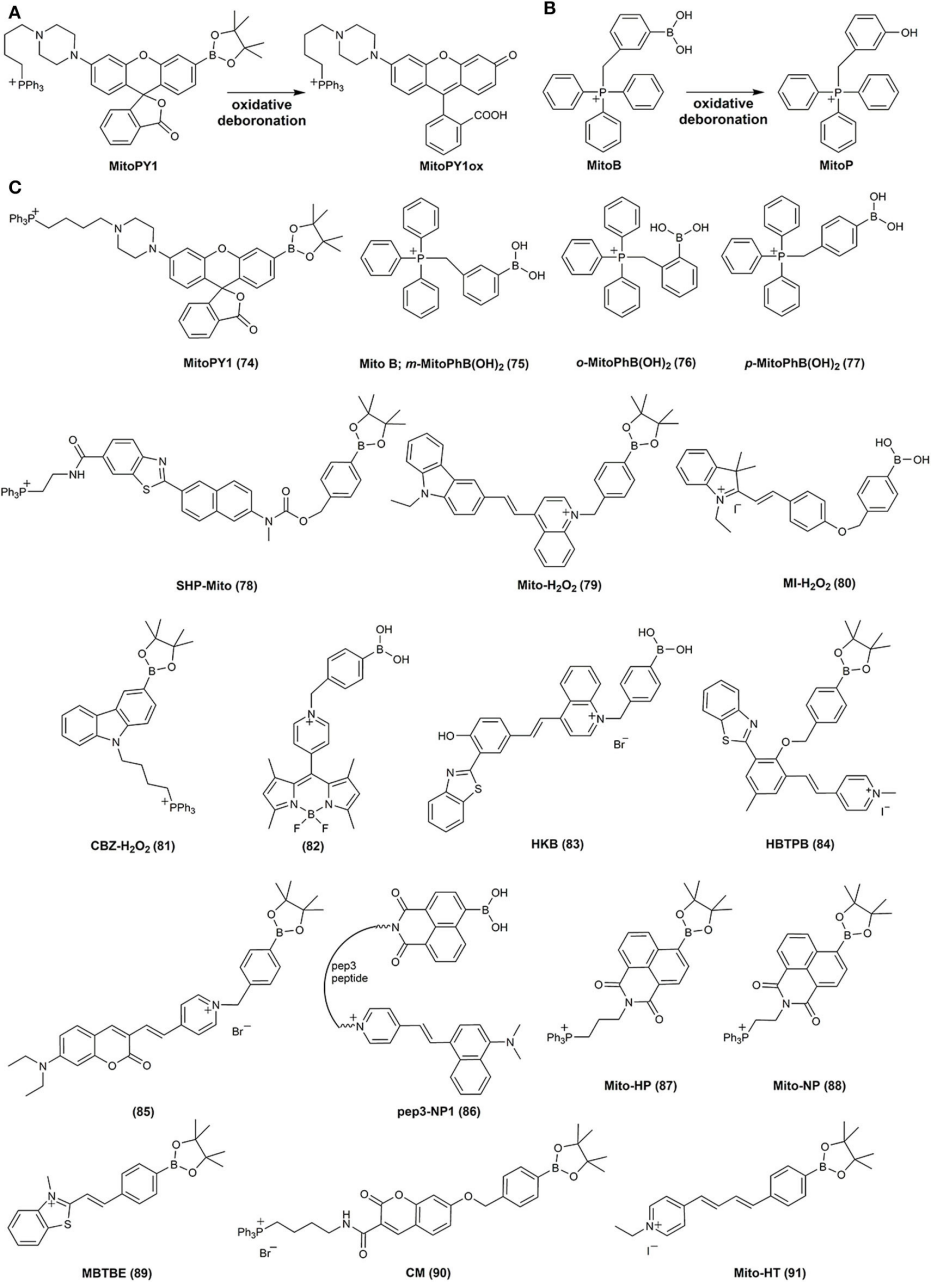
Oxidative deboronation of MitoPY1 **(A)** and MitoB **(B)**. Chemical structures of mitochondria-targeted boronate-based redox probes **(C)**.

The first reported mitochondria-targeted boronate probe, called “mitochondria-targeted Peroxy Yellow 1” (MitoPY1 (**74**), Figure 4), was a boronate derivative of a rhodol fluorescent dye linked to a triphenylphosphonium cationic moiety (TPP^+^) *via* –(CH_2_)_4_− alkyl chain (Dickinson and Chang, 2008; Dickinson et al., 2013). Linking compounds to the TPP^+^ moiety is the most common strategy to target and accumulate chemical agents in cell mitochondria (Zielonka et al., 2017b). Oxidative deboronation of MitoPY1 leads to the formation of strongly fluorescent dye, MitoPY1ox, with the reaction rate constant of 0.2 M^−1^s^−1^ (Dickinson and Chang, 2008) (Figure 4A). A different approach to detect mitochondrial H_2_O_2_ came from the Murphy's lab (Cochemé et al., 2011, 2012). The TPP^+^-conjugated simple phenylboronic acid, (3-boronobenzyl)triphenylphosphonium bromide (**75**), called MitoB (Figures 4B,C), was proposed for *in vivo* measurements of mitochondrial H_2_O_2_ production in living *Drosophila*. Both the probe and the products were detected and quantified by liquid chromatography–mass spectrometry (LC–MS)-based analyzes of the homogenates after the incubation with the probe, with the use of deuterated internal standards for most accurate quantification. It has been shown, that MitoB accumulates within mitochondria where it is oxidized to the corresponding phenol, (3-hydroxybenzyl)triphenylphosphonium cation (MitoP) (Figure 4B). The differences in H_2_O_2_ production was evaluated by the comparison of MitoP/MitoB ratios (to correct for changes in the distribution of the MitoB probe and MitoP phenolic product in the tissue under consideration). During the last decade, the MitoB probe has been applied in numerous studies on the production of mitochondrial H_2_O_2_ (Logan et al., 2014; Salin et al., 2015, 2017, 2018; Gallego-Villar et al., 2016; He et al., 2019). Also a TPP^+^-linked probe isomeric to MitoB, called *o-*MitoPhB(OH)_2_ (**76**), was shown to react with H_2_O_2_ and ONOO^−^, with the advantage of being able to distinguish those two oxidants, based on the product profiles, as discussed below (Sikora et al., 2013; Zielonka et al., 2015, 2016b). MS/MS detection parameters for both MitoB and *o-*MitoPhB(OH)_2_ probes, their oxidation products, and internal standards used are listed in Supplementary Table 1.

Several other mitochondria-targeted boronate-based fluorogenic probes were reported for the detection of H_2_O_2_ and ONOO^−^, including SHP-Mito (**78**) (Masanta et al., 2012; Kim and Cho, 2013), Mito-H_2_O_2_ (**79**) (Xu J. et al., 2016), pep3-NP1 (**86**) (Wen et al., 2015), MI-H_2_O_2_ (**80**) (Xiao et al., 2016), CBZ-H_2_O_2_ (**81**) (Zhang K. et al., 2016), HKB (**83**) (He et al., 2018), HBTPB (**84**) (Tang et al., 2018), Mito-NP (**88**) (Liang et al., 2019), MBTBE (**89**) (Shu et al., 2020b), and CM (**90**) (Weber et al., 2020) (Figure 4C, Table 3).

**Table 3:** Mitochondria-targeted boronate-based molecular probes for detection of biological oxidants.

**No**.	**Name**	**Probe**			**Oxidation product**			**Comment**	**References**
		**λ_abs_ (nm)**	**ε (M^**−1**^cm^**−1**^)**	**λ_em_ (nm); ϕ**	**λ_exc_ (nm)**	**ε (M^**−1**^cm^**−1**^)**	**λ_em_ (nm); ϕ**		
**74**	MitoPY1	489^a^ 510^a^	14,300^a^ 14,200^a^	540; 0.019^a^	510^a^	22,300	528, 0.405^a^	*k*(H_2_O_2_) ≈ 0.201 M^−1^s^−1^	Dickinson and Chang, 2008
**75**	MitoB, *m*-MitoPhB(OH)_2_	-			-		-	HPLC detection: λ_Abs_ = 268 nm or LC-MS detection^r^; *k*(H_2_O_2_) ≈ 3.8 M^−1^s^−1b^ *k*(ONOO^−^) = 1 ×10^6^ M^−1^s^−1^	Cochemé et al., 2011 Sikora et al., 2013
**76**	*o*-MitoPhB(OH)_2_	-			-		-	HPLC detection: λ_Abs_ = 268 nm or LC-MS detection^r^; *k*(ONOO^−^) = 3.5 × 10^5^ M^−1^s^−1^	Sikora et al., 2013
**77**	*p*-MitoPhB(OH)_2_	-			-		-	HPLC detection: λ_Abs_ = 268 nm or LC-MS detection; *k*(ONOO^−^) = 1 × 10^6^ M^−1^s^−1^	Sikora et al., 2013
**78**	SHP-Mito	342^d^	17,000^d^	470; 0.13^d^	383^d^	15,000^d^	545; 0.12^d^	Ratiometric probe; Two-photon probe; *k*(H_2_O_2_) ≈ 1.0–1.2 M^−1^s^−1d^	Masanta et al., 2012
**79**	Mito-H_2_O_2_	490^e^		527; 0.003^e^	376^e^		527^e^	Increase in fl. intensity	Xu J. et al., 2016
**80**	MI-H_2_O_2_	425^f^		0.0087^f^	525^f^		555; 0.11^f^	*k*(H_2_O_2_) ≈ 4.35 M^−1^s^−1f^	Xiao et al., 2016
**81**	CBZ-H_2_O_2_	340^g^		ca. 400^g^	350^g^		430^g^		Zhang K. et al., 2016
**82**		507^h^		0.002^h^	495^h^		515; 0.213^h^	Probe designed for HOCl detection	Li G. et al., 2017
**83**	HKB	564^i^		666^i^	440^i^		594^i^	Ratiometric probe; *k*(H_2_O_2_) ≈ 1.07 M^−1^s^−1i^	He et al., 2018
**84**	HBTPB	312^j^		539^j^	373^j^		669^j^	Ratiometric probe; ESIPT-based probe	Tang et al., 2018
**85**		500^k^		640^k^	500^k^		535^k^	Ratiometric probe	Shen et al., 2018
**86**	pep3-NP1	352 455^l^	9020 10,700^l^	646; 0.033^l^	455^l^		555; 0.058^l^	*k*(H_2_O_2_) ≈ 0.487 M^−1^s^−1l^; DNA-binding probe	Wen et al., 2015
**87**	Mito-HP	358^m^		543^m^	446^m^		543^m^	Increase in fl. intensity	Dai et al., 2018
**88**	Mito-NP	360^n^			460^n^		553^n^	ICT-based probe; *k*(H_2_O_2_) ≈ 3.5 M^−1^s^−1^	Liang et al., 2019
**89**	MBTBE	380^o^		0.018^o^	520^o^		569; 0.097^o^		Shu et al., 2020b
**90**	CM			400^p^	340^p^		ca. 450^p^	Ratiometric probe	Weber et al., 2020
**91**	Mito-HT	380^q^		493; 0.39^q^	395^q^		562; 0.43^q^	Ratiometric probe	Wang C. et al., 2020

a *20 mM HEPES, pH 7, 25°C*.

b *KCl medium, pH 8.0, 25°C*.

c *50 mM phosphate buffer, pH 7.4, 1 M CH_3_OH, RT*.

d *30 mM MOPS, 100 mM KCl, pH 7.4*.

e *20 mM phosphate buffer, pH 7.4, 1% DMSO, 25°C*.

f *10 mM PBS, pH 8.0*.

g *20 mM PBS, pH 7.4, 20% DMSO*.

h *20 mM PBS, pH 7.4, 1% DMSO*.

i *10 mM PBS, pH 7.0, 30% CH_3_CN*.

j *10 mM HEPES/CH_3_CN (1/1, v/v), pH 7.4*.

k *PBS/CH_3_CN, (1/9, v/v), pH 7.4*.

l *Tris-HCl, pH 7.2, 1% DMSO*.

m *10 mM PBS, pH 7.4, RT*.

n *20 mM PBS, pH 7.4*.

o *PBS : C_2_H_5_OH, 7:3, pH 7.4*.

p *PBS/CH_3_OH (52%), pH 7.4*.

q *0.01 M PBS/DMSO (5/1, v/v), pH 7.4*.

r *Molecular ions used in MS/MS-based detection are listed in Supplementary Table 1*.

It should be noted that detection of cellular oxidants requires competition with other pathways of their cellular elimination. For example, peroxiredoxins are assumed as major enzymatic scavengers of H_2_O_2_ and ONOO^−^ (Cox et al., 2009; Trujillo et al., 2017; De Armas et al., 2019; Zeida et al., 2019). Taking into account the estimated lifetime of both oxidants in the range of 0.3–1.6 ms both in the cytosolic and mitochondrial compartments (Trujillo et al., 2017), when present at 10 mM concentration, boronates would scavenge <0.001% (!) of intracellular H_2_O_2_ produced. On the other hand, at this concentration, boronates may efficiently compete for ONOO^−^ and intercept >50% of ONOO^−^. When used at a 10 μM concentration (extracellular), positively charged boronate probes are expected to reach the 100 μM level in the cytosol and ~10 mM in the mitochondrial matrix, driven by cellular plasma and mitochondrial membrane potentials (Zielonka et al., 2017b), thus demonstrating their feasibility to sensitively detect mitochondrial ONOO^−^. In case of the detection of cytosolic or mitochondrial H_2_O_2_, only a miniscule fraction of H_2_O_2_ can be directly detected by boronate probes; thus, a highly sensitive detection method would be required, and the yield of the phenolic product is expected to be a linear function of the probe's intracellular concentration. Additional factors, including the conversion of H_2_O_2_ into another oxidizing species of higher lifetime in cells and/or higher reactivity toward boronates (e.g., peroxymonocarbonate), may increase the efficiency of H_2_O_2_-induced cellular probe oxidation.

## Biological Oxidants: Hydrogen Peroxide, Peroxynitrite, Organic Hydroperoxides, and Hypochlorous Acid

Before discussing the chemical reactivity of the boronate-based redox probes toward biological oxidants, it is important to understand the basic chemical properties, biological fates, and currently used detection methods for those oxidants. In this section, we provide an overview of the chemical characteristics of H_2_O_2_, ONOO^−^, organic hydroperoxides, and HOCl, as these oxidants have been demonstrated to be formed in biological systems and to be able to oxidize the arylboronic compounds (Ainley and Challenger, 1930; Kuivila et al., 1962; Sikora et al., 2009; Lippert et al., 2011; Zielonka et al., 2012a; Michalski et al., 2014).

### Hydrogen Peroxide

Hydrogen peroxide can be generated *in vivo* in numerous biological processes, and perhaps is one of the most important redox messengers (Winterbourn, 2013; Forman et al., 2014). It can be generated directly, by enzymatic two-electron reduction of O_2_, or from dismutation of ${{\text{O}}_{2}}^{•-}$. Several enzymes produce H_2_O_2_: L-amino acid oxidase, urate oxidase, glycolate oxidase, and monoamine oxidase. The most important sources of H_2_O_2_ in cells are NADPH oxidases (Nox family of enzymes) and mitochondria. H_2_O_2_ is a strong two-electron oxidant [E°'(H_2_O_2_/H_2_O) = 1.32 V at pH 7], but due to the kinetic reasons (high activation energy barriers), it reacts slowly or not at all with most biological molecules. Due to the high p*K*_a_-value of H_2_O_2_ (p*K*_a_ = 11.6), at a physiological pH, <0.01% of hydrogen peroxide is present in the deprotonated, nucleophilic anionic form, HOO^−^. H_2_O_2_ oxidizes thiols to form sulfenic acids, but the reaction is slow in the case of most biothiols, with the family of peroxiredoxins enzymes being the major exception. H_2_O_2_ reacts rapidly with heme peroxidases (e.g., myeloperoxidase or eosinophil peroxidase) with the second order rate constants in the range of 10^7^-10^8^ M^−1^s^−1^, but these peroxidases are mostly restricted to host defense mechanisms, rather than cellular signaling. Due to the high cellular abundance and high reactivity toward H_2_O_2_, peroxiredoxins and to a smaller extent glutathione peroxidases represent the major cellular targets and enzymatic scavengers of H_2_O_2_. Another H_2_O_2_-scavenging enzyme, catalase, is mostly limited to cell peroxisomes, and its contribution to total consumption of cellular H_2_O_2_ under most conditions may be significantly lower.

Currently, only a few reliable methods for H_2_O_2_ detection and quantitation exist. In cell culture studies, extracellular H_2_O_2_ can be quantitated with the use of peroxidase-catalyzed oxidation of fluorogenic probes (currently the probe used most often for such purposes is Amplex Red, which undergoes oxidation to red fluorescent resorufin). Extracellular H_2_O_2_ also can be detected with the use of fluorogenic boronate probes, without the requirement of peroxidase, but the oxidation process is slower and a kinetic assay is required to determine the rate of probe oxidation. Intracellular H_2_O_2_ detection is still challenging (Rezende et al., 2018), and a decades-old aminotriazol-induced catalase inactivation assay remains the method of choice for quantitative estimation of the intracellular H_2_O_2_ level (Chance et al., 1979; Royall et al., 1992).

### Peroxynitrite

Peroxynitrite anion (ONOO^−^) and peroxynitrous acid (ONOOH), a Brønsted acid-base pair (p*K*_a_(ONOOH) = 6.8) (Pryor and Squadrito, 1995), are strong but unstable biological oxidants [E°'(ONOO^−^, 2H^+^/^•^NO_2_) = 1.4 V and E°'(ONOO^−^, 2H^+^/${{\text{NO}}_{2}}^{-}$) = 1.2 V at pH 7] (Koppenol et al., 1992) formed *in vivo* through the recombination of two biologically important radicals: ${{\text{O}}_{2}}^{•-}$ and ^•^NO. This reaction takes place nearly at the diffusion-controlled rate (reaction 1, *k*_1_ = 0.4–1.6 × 10^10^ M^−1^s^−1^) (Huie and Padmaja, 1993; Goldstein and Czapski, 1995; Kobayashi et al., 1995; Kissner et al., 1997), and it is believed to outcompete most other cellular routes of ^•^NO and ${{\text{O}}_{2}}^{•-}$ consumption.

$$\begin{array}{l} {{\text{O}}_{2}}^{•-}{+}^{•}\text{NO}\to \text{ONO}{\text{O}}^{-}\;\left(\text{reaction }1\right) \end{array}$$

Recently, it has been demonstrated that ONOO^−^ is also formed at physiological pH in the reaction of azanone (HNO) with molecular oxygen (reaction 2, *k*_2_ = (1.8 ± 0.3) × 10^4^ M^−1^s^−1^) (Smulik et al., 2014). However, because HNO reacts significantly faster with cellular thiols than with molecular oxygen, the biological relevance of this reaction remains to be determined.

$$\begin{array}{l} \text{HNO}+{\text{O}}_{2}\to \text{ONOOH }\left(\text{reaction }2\right) \end{array}$$

Peroxynitrite is a source of highly oxidizing radicals, which can be formed in fractional yields through a homolytic cleavage of the O–O bond of ONOOH (η ≈ 30%, reaction 3, *k*_3_ = 1.3 s^−1^ at 25°C) (Koppenol et al., 1992; Pryor and Squadrito, 1995; Ferrer-Sueta et al., 2018) or *via* the reaction of ONOO^−^ with CO_2_ (reaction 5, *k*_5_ = 2.9 × 10^4^ M^−1^s^−1^, at 25°C) (Lymar and Hurst, 1995). Peroxynitrous acid also undergoes isomerization to the nitrate anion (η ≈ 70%, reaction 4) (Pryor and Squadrito, 1995).

$$\begin{array}{l} \text{ONOOH}\to^{•}\text{OH}{+}^{•}\text{N}{\text{O}}_{2}\;\left(\text{reaction }3\right)\\ \text{ONOOH}\to \text{N}{{\text{O}}_{3}}^{-}+{\text{H}}^{+}\;\left(\text{reaction }4\right)\\ \text{ONO}{\text{O}}^{-}+\text{C}{\text{O}}_{2}\to \text{ONOOC}{{\text{O}}_{2}}^{-}\;\left(\text{reaction }5\right)\\ \text{ONOOC}{{\text{O}}_{2}}^{-}\to^{•}\text{N}{\text{O}}_{2}+\text{C}{{\text{O}}_{3}}^{•-}\;\left(\text{reaction }6\right)\\ \text{ONOOC}{{\text{O}}_{2}}^{-}\to \text{N}{{\text{O}}_{3}}^{-}+\text{C}{\text{O}}_{2}\;\left(\text{reaction }7\right) \end{array}$$

The unstable nitrosoperoxocarbonate anion (${{\text{ONOOCO}}_{2}}^{-}$) formed from the reaction between ONOO^−^ and CO_2_ (reaction 5) decays to the nitrogen dioxide radical (^•^NO_2_) and carbonate radical anion (reaction 6, η = 33% of initial ${{\text{ONOOCO}}_{2}}^{-}$) or to nitrate and CO_2_ (reaction 7, η = 67% of the initial ${{\text{ONOOCO}}_{2}}^{-}$) (Augusto et al., 2019). This reaction leads to the formation of two potent one-electron oxidants - ^•^NO_2_ and ${{\text{CO}}_{3}}^{•-}$ (Augusto et al., 2002). The production of ^•^NO_2_ makes ONOO^−^ both an oxidizing and nitrating species. The nitration of tyrosine at the 3-position causes a considerable loss of protein function and is considered as a biomarker of ONOO^−^ formation *in vivo* (Ferrer-Sueta et al., 2018). Because ^•^NO_2_ can be formed via ONOO^−^-independent pathways, including myeloperoxidase (MPO)-catalyzed oxidation of nitrite by H_2_O_2_, detection of nitrated proteins is not sufficient to conclude the involvement of ONOO^−^.

In accordance with the current knowledge of ONOO^−^ biological chemistry, only a few targets account for its consumption in biological systems. It is believed that CO_2_, peroxiredoxins, peroxidases, and a few metalloproteins are responsible for most ONOO^−^ scavenging *in vivo* (Ferrer-Sueta and Radi, 2009; Ferrer-Sueta et al., 2018).

For many years, most of the strategies for ONOO^−^ detection and quantification were depended on the reaction of probes, being the reduced fluorescent dyes with ONOO^−^-derived radicals (${{\text{CO}}_{3}}^{•-}$, ^•^NO_2_, ^•^OH). However, the detection of ROS and reactive nitrogen species with the use of those probes is non-specific (Wrona et al., 2005, 2008; Folkes et al., 2009). Moreover, such redox probes—dichlorodihydrofluorescein (DCFH) and dihydrorhodamine (DHR)—yield a corresponding fluorescent product by a free radical mechanism. It was demonstrated in a series of papers on the chemistry of DCFH and DHR, that the DCFH- and DHR-derived radicals—being products of their one-electron oxidation—react with molecular oxygen producing ${{\text{O}}_{2}}^{•-}$ (Wrona et al., 2008; Folkes et al., 2009), resulting in H_2_O_2_ production and self-propagation of the probe oxidation (Rota et al., 1999; Bonini et al., 2006).

### Organic Hydroperoxides

Organic hydroperoxides (ROOH) are compounds bearing at least one hydroperoxyl group. They can be considered structural analogs of H_2_O_2_ where one of the protons is substituted by an organic group. This class of compounds covers a wide range of chemical structures, ranging from small molecules, such as *tert*-butyl hydroperoxide, to high molecular weight compounds, including protein-bound hydroperoxides. Hydroperoxides are readily formed in biomolecules like lipids and proteins, in the presence of molecular oxygen and free radicals, or from excited-state species (Yin et al., 2011; Niki, 2014; Davies, 2016). Small-molecular-weight hydroperoxides are able to diffuse far from the site of their formation and can oxidize a wide variety of biological targets; thus, they are often considered reactive oxygen species.

The two-electron reduction potential of the organic hydroperoxyl group was estimated to be close to that of HOCl [E°'(ROOH, H_2_O/ROH) = 1.28 V] (Merenyi et al., 1994), but the reactivity of those species is largely dependent on the nature of the organic part as well as on the p*K*_a_ of the hydroperoxyl group. Isolated hydroperoxides are relatively stable and undergo slow decomposition to appropriate alcohols, but under physiological conditions they are reduced by thiols (e.g., glutathione) and ascorbate or removed by antioxidant enzymes, including peroxiredoxins and glutathione peroxidases (Peskin et al., 2010; Davies, 2016).

Hydroperoxides can be detected by titration and colorimetric methods based on iodide or ferrous ion oxidation (e.g., FOX assay), but these methods are non-specific and suitable mostly for hydroperoxides detection in simple chemical systems due to competing reactions of both assays with other biomolecules present in samples (Jessup et al., 1994; Bou et al., 2008; Michalski et al., 2014). The use of fluorescent probes like diphenyl-1-pirenyl phosphine or boronate probes is a more specific approach; however, those probes still can be oxidized by other ROS generated in the investigated system (Santas et al., 2013; Michalski et al., 2014). In the case of sterically isolated hydroperoxides (e.g., buried inside the tertiary structure of protein), there is always an uncertainty as to whether all hydroperoxyl species were scavenged by the probe, as some of the protein-derived hydroperoxides remain intact even after enzymatic digestion (Davies, 2016).

Detection of organic hydroperoxides is a challenging task. The use of fluorescent probes, rather than the onerous method requiring extraction of organic hydroperoxides from the biological matrix, enables high-throughput and kinetic studies.

### Hypochlorous Acid

Hypochlorous acid (HOCl) is another important ROS. This potent oxidizing and chlorinating agent is generated *via* the reaction of H_2_O_2_ with chloride ions, catalyzed by the heme enzyme MPO (Davies et al., 2008). The p*K*_a_ of HOCl is equal to 7.5 (Morris, 1966); thus, both HOCl and the hypochlorite anion (OCl^−^) are present at nearly equimolar concentrations at physiological pH.

HOCl is a strong two-electron oxidizing [E°'(HOCl, H_2_O/Cl^−^) = 1.28 V] (Arnhold et al., 2006) and chlorinating agent. It reacts rapidly with nucleophiles such as thiols and amines; thus, cysteine residues in proteins and glutathione (GSH) are the main targets for cellular HOCl (Davies et al., 2008). Due to the high reactivity of HOCl toward thiols and amines, its intracellular detection is difficult. In most cases, the probe would be able to intercept only a small fraction of the total amount of HOCl produced and/or react with the products of the interaction of HOCl with biomolecules.

There are numerous reported examples of fluorescent probes designed for the detection of HOCl utilizing the strong oxidizing character of HOCl and specific redox reactions of HOCl (Zhang et al., 2018; Wu D. et al., 2019). Nonetheless, in most cases, the chemistry of those probes has not been fully characterized, and their biological validation should include an independent and established method of HOCl detection. One of the reasonable approaches for selective monitoring of HOCl in biological systems can be the detection of products of HOCl-induced chlorination. The conversion of a hydroethidine probe to 2-chloroethidium represents a detection strategy, utilizing both the oxidizing and chlorinating potential of HOCl (Maghzal et al., 2014; Talib et al., 2016). It has been shown that boronate probe peroxy-caged luciferin (PCL-1) reacts with HOCl, forming a phenolic product that further undergoes chlorination producing 7′-chloroluciferin (Kalyanaraman et al., 2016; Zielonka et al., 2016a). It is well-established that arylboronates react stoichiometrically with HOCl to produce phenolic products (Sikora et al., 2009). Although the use of boronic probes for HOCl monitoring in cellular studies is not without caveats, due to their moderate reactivity toward HOCl (^2^*k* ~ 10^4^ M^−1^s^−1^) and the high reactivity of HOCl toward endogenous thiols and amines, boronate redox probes can be used in cell-free-based screening assays for MPO inhibitors. Also, boronate probes may be able to intercept HOCl in the cell culture/assay medium, where the lifetime of HOCl may be significantly higher than inside the cells.

## Chemical Reactivity of Boronates Toward Biological Oxidants

### Biological Oxidants Reported to React With Arylboronates

Since the first reports on the development of boronate-based redox probes (Lo and Chu, 2003; Chang et al., 2004; Akhavan-Tafti et al., 2005), numerous authors have claimed the selectivity of this class of probes toward H_2_O_2_. However, analysis of the scientific literature on the chemical reactivity of arylboronates clearly points to the ability of other nucleophilic oxidants to convert them to corresponding phenols. In 1930, phenylboronic acid was reported to react with H_2_O_2_, forming phenol (Ainley and Challenger, 1930). However, the same paper also showed that phenylboronic acid is oxidized to phenol in chlorine or bromine water, and it was proposed that the reacting oxidant is HOCl/HOBr (Ainley and Challenger, 1930). The oxidation of phenylboronic acids by HOCl and HOBr anions also was reported in 1962 (Kuivila et al., 1962). It was shown that arylboronates are substrates for flavin-containing cyclohexanone oxygenase, and flavin hydroperoxide was proposed as the reactive oxidant (Branchaud and Walsh, 1985). In 2009, another biologically important oxidant, ONOO^−^, was shown to react directly and rapidly with arylboronates, yielding corresponding phenols as the main products (Sikora et al., 2009). The second-order rate constants for the reaction of boronates with ONOO^−^, HOCl, and H_2_O_2_ were determined at pH 7.4, and it was demonstrated that ONOO^−^ reacts with boronic acids nearly a million times faster (*k* ~ 10^6^ M^−1^s^−1^) than H_2_O_2_ (*k* ~ 1 M^−1^s^−1^) and more than 100 times faster than HOCl (*k* ~ 10^4^ M^−1^s^−1^) (Sikora et al., 2009). This high reactivity of arylboronic acids toward ONOO^−^, compared with H_2_O_2_, makes them potential probes for intracellular detection of ONOO^−^. Based on the substrate consumption study and the analysis of phenols formation yield, arylboronates were concluded to react with peroxynitrite at a 1:1 stoichiometry, yielding the corresponding phenol as a major product (η = 80–85%) (Sikora et al., 2009). Also, the reaction of peroxynitrite with arylboronates was proposed to lead to the formation of radical transient species and radical-derived minor products (η = 15–20%) (Sikora et al., 2011). Both HOCl and H_2_O_2_ react stoichiometrically with boronates, yielding the corresponding phenols with a yield close to 100%. When used at excess, HOCl has been shown to cause a rapid decrease in the yield of phenol, which can be attributed to the chlorination of phenol by excess HOCl. The oxidation of boronates was observed in systems where ^•^NO and ${{\text{O}}_{2}}^{•-}$ are co-generated, producing peroxynitrite *in situ* (Sikora et al., 2009). Also, it was verified that ^•^NO_2_ does not oxidize boronates to phenols (Sikora et al., 2009).

**Scheme 4:**
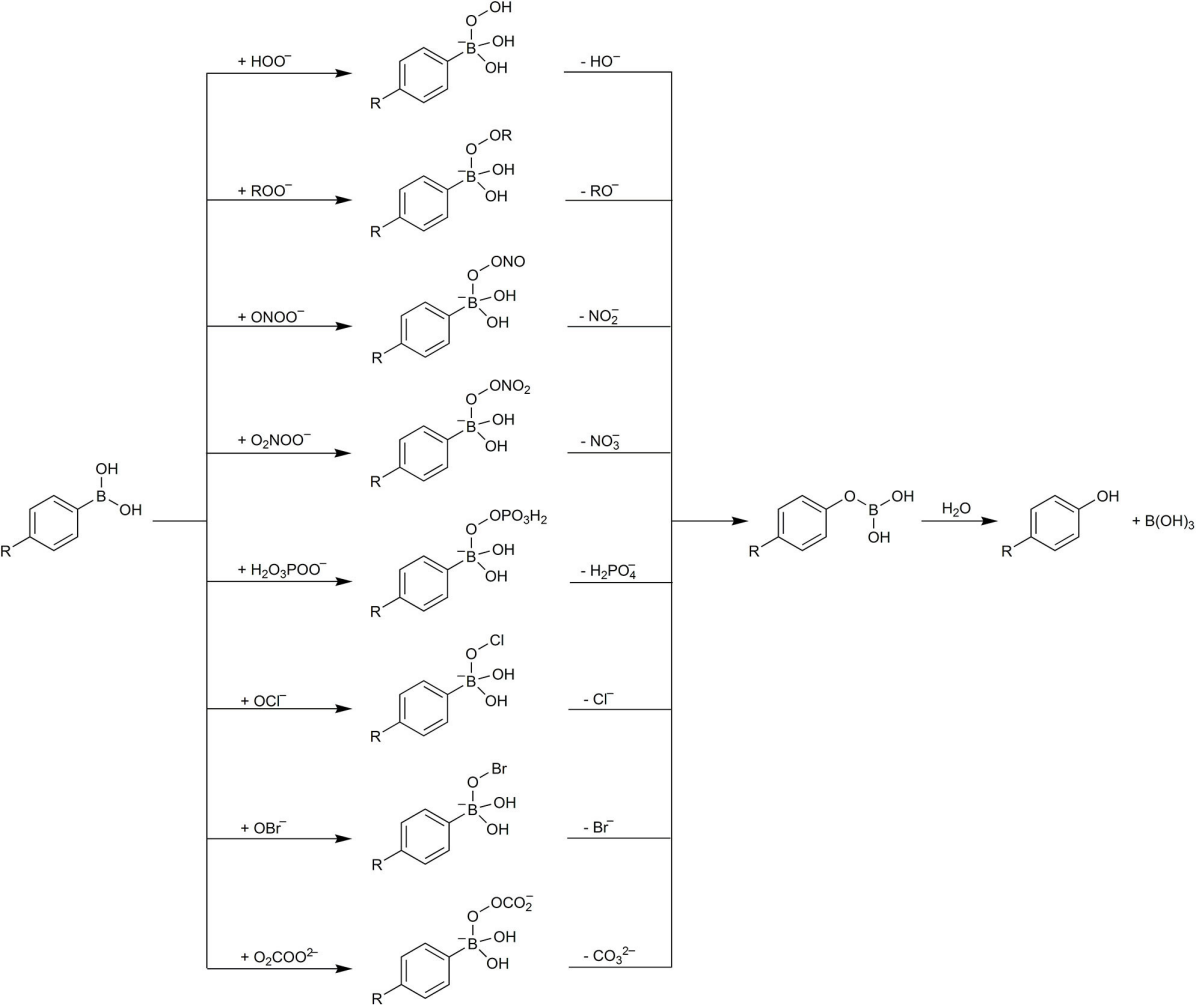
The general mechanism of boronate oxidation by anionic peroxides and the anions of hypohalous acids.

The proposed mechanism of the simple phenylboronates reaction with ONOO^−^, OCl^−^, and H_2_O_2_ is quite general and can be applied to other acidic hydroperoxides and hypohalous acids as well as to other boronates, including boronate-based profluorescent probes, erroneously described in the literature as selective for H_2_O_2_. Over the years, more oxidants have been demonstrated to be able to oxidize boronic compounds, including amino acid hydroperoxides (Michalski et al., 2014), peroxynitrate (O_2_NOO^−^) (Huang et al., 2019), peroxymonophosphate (O_3_POO^3−^) (LaButti and Gates, 2009), and peroxymonocarbonate (O_2_COO^2−^) (Truzzi and Augusto, 2017). Boronic acids and esters are organic compounds possessing trivalent, an sp^2^-hybridized boron atom that is linked to one alkyl or aryl substituent and two hydroxyl or ester groups. They are mild and hard Lewis acids that can easily coordinate hard nucleophilic bases. The p*K*_a_-value for most arylboronic acids is in the range of 7–9, depending on the structure. The nucleophilic addition of peroxides (HOO^−^, ROO^−^, ONOO^−^, O_2_NOO^−^, O_3_POO^3−^, O_2_COO^2−^) or hypohalite anions (OCl^−^, OBr^−^) to the boronate functional group is a facile reaction and the first step of the oxidation reaction. Based on available experimental and computational data, a general mechanism for arylboronates oxidation can be proposed (Scheme 4). The reaction occurs with the formation of the adduct of anionic oxidant to the boronic functional group, followed by the heterolytic cleavage of the O–O or O–halogen bond, resulting in the elimination of an anionic leaving group and formation of phenoxyboronic acid intermediate. This intermediate undergoes hydrolysis to form phenolic product and boric acid (Scheme 4). Isotope tracking experiments indicate that the oxygen atom in the phenolic product derives from the oxidant and not from the solvent (Rios et al., 2020).

The reported differences in the second-order rate constants for boronate oxidation by H_2_O_2_, HOCl, and ONOO^−^ at pH 7.4 (Sikora et al., 2009) can be partially explained by the differences in their p*K*_a_-values: 11.7 (H_2_O_2_), 7.5 (HOCl), and 6.8 (ONOOH). The calculated fractions of the boronate-reactive anionic forms, HOO^−^, OCl^−^ and ONOO^−^, at pH 7.4 are 0.005, 46, and 80%, respectively. Other factors, including the type of the anionic leaving group and the extent of its solvation, also should be considered.

The effect of changes of pH on the reactivity of biological oxidants with boronates is complex and involves the acid-base equilibria of both the probe and the oxidant. Further studies are required, but based on the authors' experiences, two general assumptions can be made: (i) the higher the pH, the higher the amount of deprotonated oxidant is present, resulting in a faster oxidation process; and (ii) the higher the pH, the higher the fraction of the boronate probe is present in the basic form, with the boron atom in sp^3^ hybridization, resulting in slower reaction kinetics. It can be anticipated that, for each specific probe and oxidant, there exists an optimal pH, with the highest rate constant of the oxidation reaction. Furthermore, for fluorescence-based detection, phenolic products may undergo acid-based equilibration within the physiological pH range, which will be reflected in the pH-dependent fluorescence signal.

### Mechanism of Oxidation of Boronates by ONOO^−^

Product analyses and substrate consumption data, in combination with the kinetic data presented in the first two studies on the ONOO^−^-induced boronate oxidation (Sikora et al., 2009; Zielonka et al., 2010), indicate that all three oxidants tested (H_2_O_2_, HOCl, and ONOO^−^) react with boronates directly and stoichiometrically (with 1:1 stoichiometry), yielding the corresponding phenols as the sole (in the case of H_2_O_2_, HOCl) or major (yield of about 80–90% in the case of ONOO^−^) product. The formation and identity of other, minor products formed in the reaction of boronates with ONOO^−^ implicated the occurrence of a second, free radical pathway, involving the formation of phenoxyl, phenyl, and ^•^NO_2_ radicals. This minor radical pathway was studied and described in detail in Sikora et al. (2011). The electron paramagnetic resonance (EPR) spin-trapping technique was used to trap and characterize the radical transient products formed in the reaction between boronic acids and peroxynitrite. The formation of phenyl radicals in the studied reaction was unequivocally confirmed with the use of two spin traps, 2-methyl-2-nitrosopropane (MNP) and 5-diethoxyphosphoryl-5-methyl-1-pyrroline-N-oxide (DEPMPO), based both on the spectral signatures and the inhibitory effects of 2-propanol, the scavenger and reductant of phenyl radical, on the yield of the spin adduct. Analogous spin-trapping experiments using H_2_O_2_ as the oxidant did not show formation of any radical intermediates, in agreement with the established yield of the phenolic product.

**Scheme 5:**
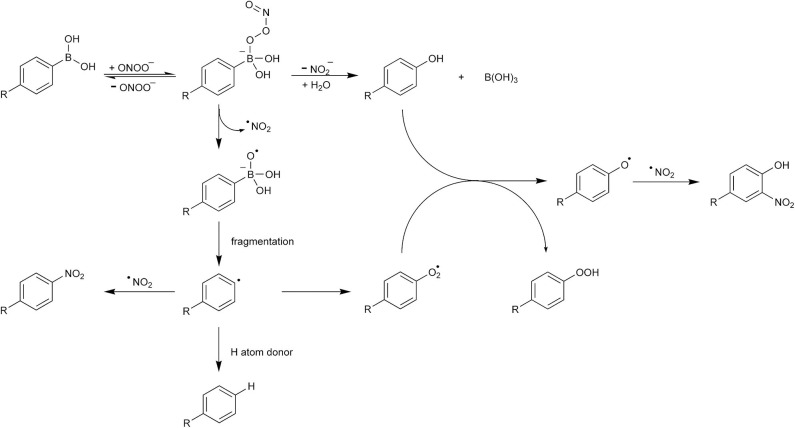
Mechanism of peroxynitrite reaction with phenylboronic acids.

Based on these observations, the mechanism of ONOO^−^-induced boronate (PhB(OH)_2_) oxidation was proposed (Scheme 5) (Sikora et al., 2011). The initial step of that reaction is the nucleophilic addition of ONOO^−^ to the electrophilic boron atom in PhB(OH)_2_, resulting in the formation of an anionic adduct. This adduct undergoes further transformation into products *via* two mechanisms. In the major pathway, the O–O bond in the adduct undergoes heterolytic cleavage with the elimination of the nitrite anion, resulting in the formation of phenol and boric acid. In the minor pathway, the O–O bond in the adduct undergoes homolytic cleavage, resulting in the formation of ^•^NO_2_ and PhB(OH)_2_O^•−^ transient radical products. The phenyl radical (Ph^•^) can be subsequently formed *via* the fragmentation of the PhB(OH)_2_O^•−^ radical anion or of its protonated form, the ${{\text{PhB(OH)}}_{3}}^{•}$ radical. The latter can be regarded as an adduct of the phenyl radical Ph^•^ to boric acid (B(OH)_3_) and should be extremely unstable. Indeed, according to the reported results of density-functional theory quantum mechanical calculations, the energy barrier for the fragmentation of the PhB(OH)_2_O^•−^ radical anion leading to the formation of phenyl radical is very low (12–17 kJ/mol) (Sikora et al., 2011) and the dissociation of the ${{\text{PhB(OH)}}_{3}}^{•}$ radical into Ph^•^ and B(OH)_3_ is barrierless. Once formed, the phenyl radical may undergo subsequent reactions with hydrogen atom donors, molecular oxygen, or the ^•^NO_2_ radical (Scheme 5). The latter reaction, which may occur within the solvent cage, results in the formation of a stable ONOO^−^-specific nitrobenzene product, PhNO_2_. Rapid reaction of Ph^•^ with O_2_ results in the formation of the highly oxidizing phenylperoxyl radical PhOO^•^, which can oxidize phenols, yielding phenoxyl radical PhO^•^ and products derived from it. Inhibitory effects of hydrogen atom donors (e.g., 2-propanol) and radical scavengers (e.g., NADH, GSH, ascorbic acid) on the formation of phenoxyl radical-derived nitrophenolic product(s) confirmed that the phenoxyl radical is formed as the secondary transient radical species.

It has been proposed that the ONOO^−^-specific products' profile of boronate oxidation reaction can be used in chemical, enzymatic, and cellular systems as a “peroxynitrite fingerprint” to confirm the presence of this oxidant. This is a significant advantage over the detection of protein nitration, which may occur via ONOO^−^-independent pathways, as mentioned above. The peroxynitrite-specific products of *o-*MitoPhB(OH)_2_ and PCL-1 oxidation have been detected in extracts of activated macrophages (Zielonka et al., 2016a,b). The relative contribution of the radical pathway during oxidation of boronate compounds by ONOO^−^ is, however, dependent on the chemical structure of the boronate. For example, it has been reported that the Fl-B probe undergoes conversion to the fluorescein product with a 99% yield (Rios et al., 2016). Therefore, for each new boronate probe developed for ONOO^−^ detection, the reaction mechanism should be determined and product distribution quantified.

**Scheme 6:**
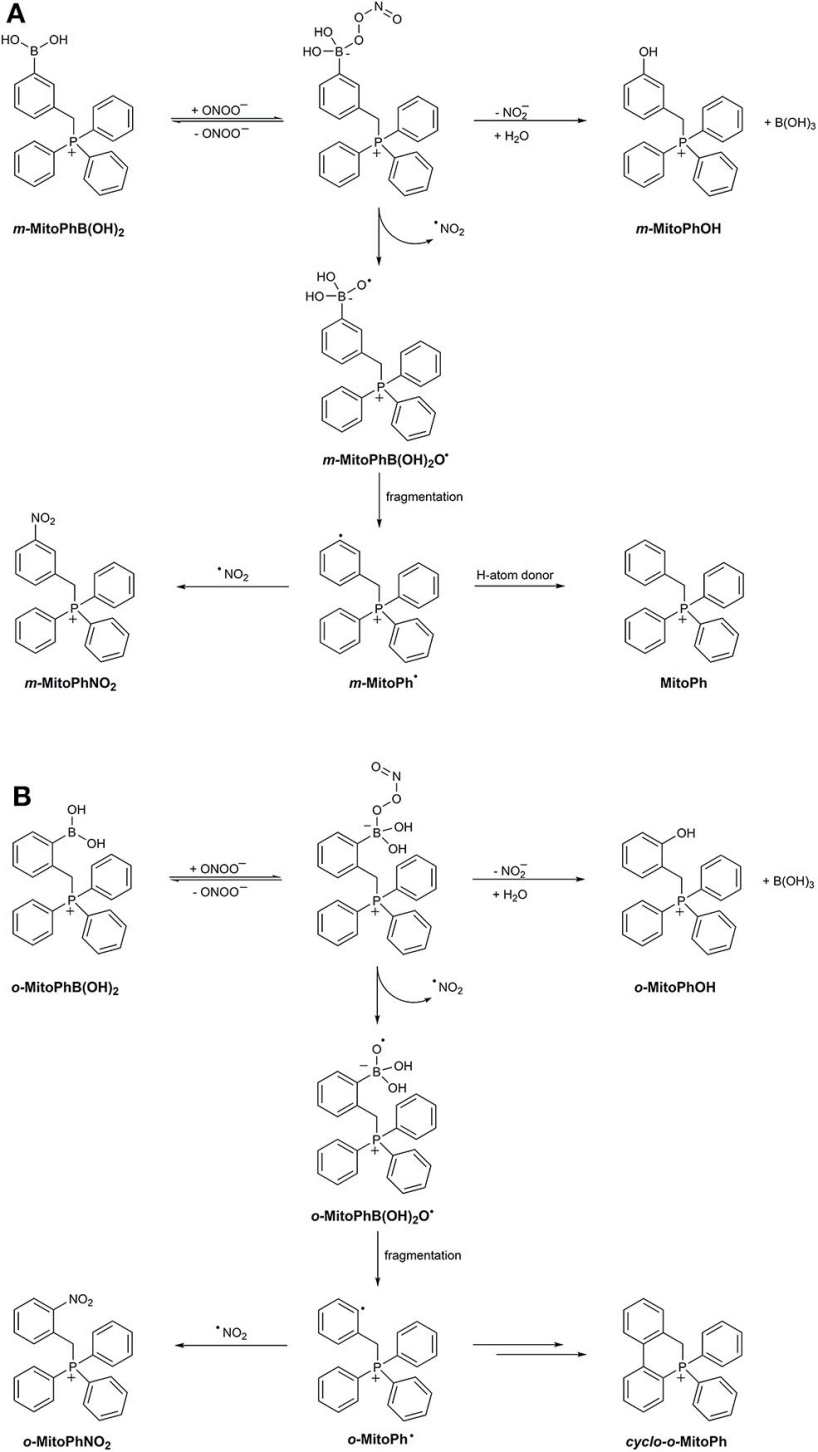
Mechanism and products of the reaction between peroxynitrite and **(A)** *m-*MitoPhB(OH)_2_ or **(B)** *o-*MitoPhB(OH)_2_ probe.

The mechanism of ONOO^−^-derived boronate oxidation was further explored in studies on the reaction of the mitochondria-targeted probe MitoB (*m-*MitoPhB(OH)_2_) and its isomers *o-*MitoPhB(OH)_2_ and *p-*MitoPhB(OH)_2_ with ONOO^−^ (Sikora et al., 2013; Zielonka et al., 2015, 2016b; Rios et al., 2020). Similar to simple arylboronates, MitoB and its *para-* and *ortho-*isomers react rapidly with ONOO^−^ with the formation of the corresponding phenolic products at 90% reaction yield. The determined second-order rate constants for the reaction of ONOO^−^ with MitoPhB(OH)_2_ isomers at pH 7.4 are equal to (3.5 ± 0.5) × 10^5^, (1.0 ± 0.1) × 10^6^, and (1.0 ± 0.1) × 10^6^ M^−1^s^−1^ for *ortho-, meta-*, and *para-*isomers, respectively (Sikora et al., 2013). All three isomers react with ONOO^−^ with the formation of additional, minor products *via* the radical pathway. EPR spin-trapping experiments showed that the phenyl radicals formed from the reaction between ONOO^−^ and *m-*MitoPhB(OH)_2_ or *p-*MitoPhB(OH)_2_ but not from the *ortho-*isomer could be trapped using the MNP and DEPMPO spin traps. In the case of *para-* and *meta-*isomers, the dominant minor product formed from the radical pathway in the presence of phenyl radical reductant, 2-propanol, is MitoPhH, formed by hydrogen atom abstraction from 2-PrOH by MitoPh^•^ radicals. In case of the *ortho-*isomer, not only was the EPR spin trapping of the phenyl radical unsuccessful, but also the yield of MitoPhH in the presence of 2-PrOH was significantly lower. Nevertheless, for all three isomers, the additional minor nitration product, MitoPhNO_2_, was detected (Sikora et al., 2013). This product was also detected in macrophages activated to produce ONOO^−^ in the presence of the *o-*MitoPhB(OH)_2_ probe (Zielonka et al., 2015, 2016b). Mass spectrometric-based investigation on the products formed in the reaction between *o-*MitoPhB(OH)_2_ and ONOO^−^ led to the conclusion that the transient phenyl radical *o-*MitoPh^•^ predominantly undergoes rapid intramolecular cyclization to form a peroxynitrite-specific product, cyclo-*o-*MitoPh (9,10-dihydro-9,9-diphenyl-9-phosphoniaphenanthrene) (Scheme 6B), in 10.5% yield while the nitrobenzene-type product, *o-*MitoPhNO_2_, is formed in only 0.5% yield (Zielonka et al., 2016b).

As with simple arylboronates, the formation of the observed products of the ONOO^−^ reaction with *o-*MitoPhB(OH)_2_ can be rationalized assuming the mechanism where the ONOO^−^ adduct to the electrophilic boron atom decays on two pathways: *via* heterolytic or homolytic cleavage of the O–O bond (Scheme 6) (Sikora et al., 2013). The major, heterolytic cleavage pathway leads to the formation of the corresponding phenol, MitoPhOH, and ${{\text{NO}}_{2}}^{-}$. The minor, homolytic cleavage pathway results in the formation of a radical anion, MitoPhB(OH)_2_O^•−^, and ^•^NO_2_. Subsequent fragmentation of MitoPhB(OH)_2_O^•−^ to MitoPh^•^ and its further reactions (including intramolecular cyclization in the case of *o-*MitoPh^•^, Scheme 6B) result in the formation of ONOO^−^-specific minor products (Sikora et al., 2013; Zielonka et al., 2016b). The proposed mechanism has been further corroborated by tracking the O−18 atoms in the products formed during the reaction between *o-*MitoPhB(OH)_2_ and ON^18^O^18^O^−^ (Rios et al., 2020). Relative quantitative analyses of the non-radical and radical-derived products of MitoPhB(OH)_2_ oxidation give insight into the kinetics of both pathways. The ratio of the rate constants of the radical and non-radical pathways *k*_rad_/*k*_non−rad_ can be estimated from the plot of sum of the minor products vs. the amount of major product. The determined values of those ratios were equal to 0.1, 0.1, and 0.07 for *ortho-, meta-*, and *para-*isomers, respectively (Sikora et al., 2013).

### Boronate-Based Molecular Probes in the Studies of HNO Reactivity

Boronate-based molecular probes have emerged as a valuable tool in studies on HNO reactivity. In 2014, coumarin-7-boronic acid (CBA, **26**) and *p*-MitoPhB(OH)_2_ (**77**) probes were used to determine the chemical identity of the product of the HNO reaction with O_2_ in aqueous solutions at physiological pH (Smulik et al., 2014).

HNO, commonly known as nitroxyl (IUPAC recommended name: azanone), formally is the protonated product of the one-electron reduction of nitric oxide. This reactive triatomic molecule remains one of the most enigmatic reactive nitrogen species, as its biological chemistry and physiological activity are still not completely understood. For many years, one of the most intriguing aspects of HNO chemistry was its reaction with O_2_. It has been reported in the literature that the azanone anion (NO^−^) reacts rapidly with molecular oxygen (*k* = 2.7 × 10^9^ M^−1^s^−1^), forming ONOO^−^ (Donald et al., 1986; Shafirovich and Lymar, 2002) (reaction 8).

$$\begin{array}{l} \text{N}{\text{O}}^{-}+{\text{O}}_{2}\to \text{ONO}{\text{O}}^{-}\;\left(\text{reaction }8\right) \end{array}$$

Despite several studies on the reaction of HNO with O_2_, the chemical identity of the oxidant that is formed in this reaction has been controversial (Miranda et al., 2001, 2002; Kirsch and De Groot, 2002). The possibility of ONOO^−^ formation has been proposed (Kirsch and De Groot, 2002), but it has also been questioned (Miranda et al., 2001, 2002). This was due, in part, to the lack of appropriate molecular probes that directly react with ONOO^−^ with the formation of ONOO^−^-specific products. As has been discussed, several boronate compounds have shown that their reaction with peroxynitrite leads to the formation of a major phenolic product and several peroxynitrite-specific minor products (Sikora et al., 2009, 2011, 2013; Smulik et al., 2014; Zielonka et al., 2015, 2016a,b; Rios et al., 2020). The generation of ONOO^−^ in the reaction of HNO with O_2_ in aqueous solutions at pH 7.4 was confirmed based on the oxidation of the boronate probes used and the formation of ONOO^−^-specific products in the solutions of HNO donors (Smulik et al., 2014). The use of a set of HNO scavengers of known reactivity and the competition kinetics method allowed the second order rate constant for the HNO reaction with O_2_ [*k* = (1.8 ± 0.3) × 10^4^ M^−1^s^−1^] to be determined (Smulik et al., 2014). This value is two times higher than previously reported (*k* = 8 × 10^3^ M^−1^s^−1^) (Liochev and Fridovich, 2003) and ~5 times higher than the value proposed by Miranda et al. (2003) (*k* = 3 × 10^3^ M^−1^s^−1^). The use of boronate redox probes, in combination with a competition kinetics approach, was proposed to study the reactivity of HNO toward its scavengers, and the determination of the kinetics of HNO reaction with selected thiols was reported.

The same methodology, in combination with the use of the resorufin-based boronate probe, PC1 (**15**), was also used in a study on the reactivity of HNO toward selected scavengers (Smulik-Izydorczyk et al., 2017). The kinetic method was based on two parallel HNO reactions: one with the studied scavenger and the other with molecular oxygen [*k* = (1.8 ± 0.3) × 10^4^ M^−1^s^−1^ (Smulik et al., 2014)]. The latter led to the formation of ONOO^−^, which was detected fluorometrically with the use of the PC1 probe.

Recently, a kinetic study on HNO liberation from Piloty's acid derivatives showed that boronate-based molecular probes can be a useful tool for the characterization of HNO donors (Smulik-Izydorczyk et al., 2019). Again, the indirect method of HNO detection was used, based on its reaction with O_2_ to form peroxynitrite, and quantitation using boronate-based redox probe PC1.

### Fluorescence-Based Monitoring of H_2_O_2_ and ONOO^−^ Formed *in situ* in Enzymatic and Cellular Systems

One of the first reported fluorogenic boronate probes, PF1 (**3**), was shown to undergo intracellular oxidation to a green-fluorescent product upon exposure of live cells to H_2_O_2_ (Chang et al., 2004). This report provides a proof of principle for the ability of boronate-based probes to respond to H_2_O_2_ in a cellular milieu. Building on this observation, a series of mono-boronated probes [e.g., PG1 (**16**)] have been synthesized and shown to respond to endogenously produced H_2_O_2_, demonstrating the potential of boronate-based redox reporters as a new generation of probes for redox biology (Miller et al., 2007). CBA (**26**) was the first boronate-based profluorescent probe applied for the real-time monitoring of ONOO^−^ formation in an enzymatic system (Zielonka et al., 2010) and in cultured cells (Zielonka et al., 2012b). Similar to simple arylboronates, CBA has been shown to react rapidly and stoichiometrically with ONOO^−^, yielding fluorescent 7-hydroxycoumarin (COH) as the major product (η ≈ 83%) and ONOO^−^-specific minor products: coumarin and 7-nitrocoumarin (Smulik et al., 2014). The observed reactivity of CBA toward biological oxidants is similar to what had been published for arylboronates: the second-order rate constants for the reaction of CBA with ONOO^−^ and H_2_O_2_ are equal to 1.1 × 10^6^ and 1.5 M^−1^s^−1^, respectively (Zielonka et al., 2010). The CBA probe was used to demonstrate the formation of ONOO^−^ as the product of the reaction of ${{\text{O}}_{2}}^{•-}$ and ^•^NO, when used at different ratios (Zielonka et al., 2010) and as a product of the reaction of HNO with O_2_ (Smulik et al., 2014). Following the CBA- and FlAmBE (**22**)-based detection of ONOO^−^ in RAW 264.7 macrophages stimulated to coproduce ${{\text{O}}_{2}}^{•-}$ and ^•^NO (Zielonka et al., 2012b), numerous boronate redox probes were developed and reported for the detection of cellular ONOO^−^ (Yu et al., 2012; Kim J. et al., 2014; Rios et al., 2016; Sedgwick et al., 2017; Murfin et al., 2019; Weber et al., 2020). CBA was also used in combination with the immuno-spin trapping technique to demonstrate ONOO^−^-mediated protein radical formation in microglial cells exposed to lipopolysaccharide (Kumar et al., 2014).

### Fluorescence-Based Monitoring of Amino Acid and Protein-Bound Hydroperoxides

**Scheme 7:**
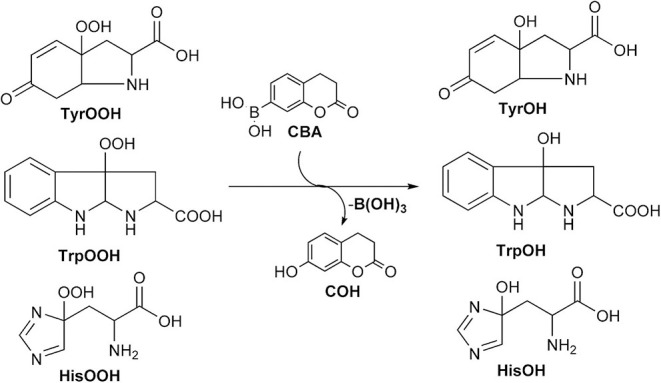
Oxidation of CBA to COH by amino acid-derived hydroperoxides.

Boronate probes have been also proposed for the detection of amino acid, peptide, and protein hydroperoxides (Michalski et al., 2014). CBA (**26**) has been shown to react with enzymatically generated tyrosyl hydroperoxide to form the fluorescent COH product (Scheme 7).

Although the stoichiometry of the reaction between boronate probes and amino acid, peptide, and protein hydroperoxides has never been directly measured due to the lack of authentic standards of the corresponding hydroperoxides, it is reasonable to assume a 1:1 stoichiometry by analogy with the structurally related H_2_O_2_ and other tested oxidants (Sikora et al., 2009; Zielonka et al., 2010). The developed fluorometric assay for amino acid hydroperoxides has been adapted to a multi-well plate format and compared to the FOX assay, revealing several advantages, including a one-step detection process, a possibility of real time measurements, and superior assay quality as assessed by the Z' parameter (Michalski et al., 2014). Further studies using photochemically generated singlet oxygen enabled production of tyrosyl, tryptophan, and histidine hydroperoxides, and their reactivity toward the boronate probe was tested. These hydroperoxides have been shown to react with the CBA probe with rate constants at least 10 times higher as compared with H_2_O_2_. Finally, CBA has been successfully used for the detection of protein hydroperoxides generated on BSA and lysozyme and with those produced in lysates from cells exposed to visible light and the singlet oxygen-generating photosensitizer Rose Bengal. Subsequent studies on the reaction of boronated probes with amino acid hydroperoxides have shown that the high concentration of biothiols can affect the signal from the boronate probe (Debowska et al., 2016). The presence of a 0.3–3 mM concentration of GSH significantly decreased the fluorescence signal from probe oxidation (present at 10 μM), while 10 mM GSH completely abolished probe oxidation, indicating a direct reaction of GSH with tyrosyl hydroperoxide. On this basis, it can be concluded that detection of cellular hydroperoxides requires a relatively high concentration of the boronate probe to enable efficient competition with cellular scavengers of hydroperoxides. The CBA probe seems well-suited for this specific purpose due to its relatively high water solubility and superior chemical stability (Zielonka et al., 2010; Michalski et al., 2014).

### Bioluminescence-Based Detection of Biological Oxidants

**Scheme 8:**
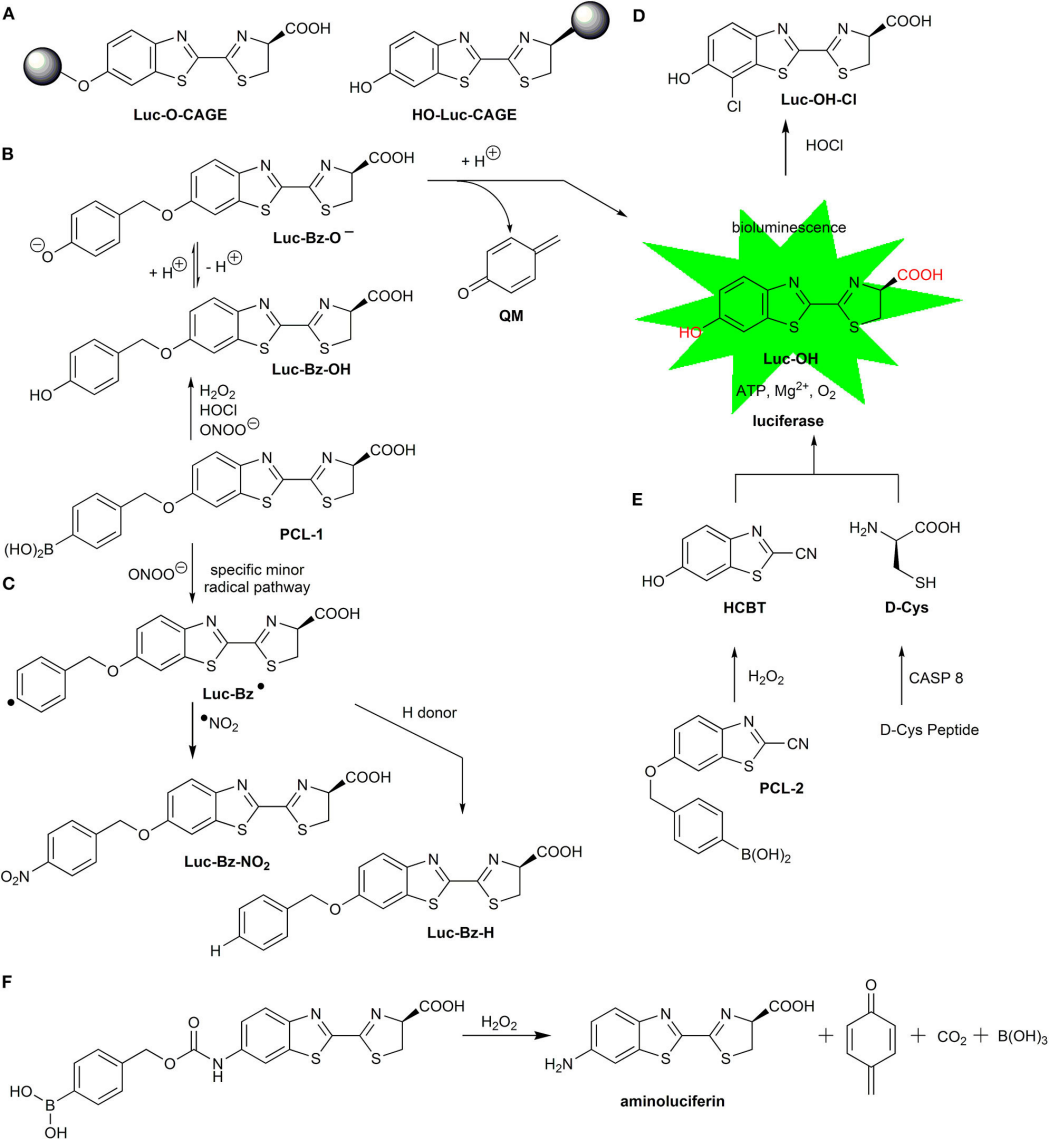
Luminogenic boronate probes and their reactions with biological oxidants. **(A)** Mode of masked luciferin. **(B)** Reaction of the PCL-1 probe with H_2_O_2_, HOCl, and ONOO^−^ leading to luciferin after the release of QM. **(C)** Radical pathway to peroxynitrite-specific products. **(D)** Formation of 7′-chloroluciferin. **(E)** Formation of firefly luciferin *in situ* after release of 6-hydroxy-2-cyanobenzothiazole and D-cysteine. **(F)** Oxidation of boronic acid-caged aminoluciferin.

Bioluminescent imaging is commonly used for sensitive monitoring of various biomolecular processes in cells and living animals. The popularity of bioluminescence assays in biomedical research resulted in significant progress in the syntheses of luminogenic probes based on the firefly luciferin skeleton (Luc-OH, Scheme 8). The blocking (also referred to as “caging”) of the hydroxyl or carboxyl group (Scheme 8A) by chemical systems, which are capable of reacting specifically with the analyte of interest, makes recognizing the probe (Luc-O-CAGE, HO-Luc-CAGE) by luciferase impossible and thus blocks the formation of luminescence. The release of Luc-OH as a result of the dislodgement of the blocking group occurs during or after recognition of the analyte. Then, luciferase interacts with Luc-OH and the required co-factors, and the bioluminescence signal is observed and recorded using luminescence reader or imaging system.

Several luciferin-based oxidant-sensitive probes have been developed and reported (Van De Bittner et al., 2010; Wu et al., 2014; Kojima et al., 2015; Chen et al., 2017). Among those probes, all boronate-based luciferin derivatives contain self-immolative boronobenzyl moieties. Recently, a luciferin derivative, with the hydroxyl group directly substituted with the boronate group, was reported and shown to respond to activated macrophages (Szala et al., 2020). One of the first pro-luciferin bioluminescent probes designed for imaging H_2_O_2_ in live animals was peroxy-caged luciferin, PCL-1 (Van De Bittner et al., 2010) (Scheme 8). The second order rate constant for the reaction between PCL-1 and H_2_O_2_ was determined to be equal to 3.8 ± 0.3 M^−1^s^−1^. In 2014, boronic acid-caged aminoluciferin (CAL) was synthesized and applied as a probe for real-time detection of H_2_O_2_ *in vitro, in cellulo*, and *in vivo* (Wu et al., 2014). In both PCL-1 and CAL, the boronobenzyl moiety is oxidized to intermediate phenol, which subsequently releases luciferin (Schemes 8B,F) or aminoluciferin Luc-NH_2_, respectively, *via* a self-immolative reaction mechanism.

Follow-up studies determined that the PCL-1 probe also can be oxidized by ONOO^−^ [*k* = (9.8 ± 0.3) × 10^5^ M^−1^s^−1^] (Sieracki et al., 2013). Similar to simple arylboronates, the reaction of PCL-1 with ONOO^−^ proceeds *via* two pathways. The major pathway is the same as for H_2_O_2_ and involves the formation of the phenolic intermediate (Luc-Bz-OH), which after elimination of QM yields Luc-OH as the stable end product. Rapid HPLC analyses enabled the detection of the primary phenolic intermediate formed upon the reaction of PCL-1 with different oxidants (Zielonka et al., 2016a). The minor pathway of the reaction of PCL-1 with ONOO^−^ produces nitrated and reduced products, Luc-Bz-NO_2_ and Luc-Bz-H, respectively (Scheme 7C). The formation of phenyl-type radical Luc-Bz^•^ during oxidation of PCL-1 by ONOO^−^ was confirmed by EPR spin trapping combined with liquid chromatography-mass spectrometry (LC-MS)-based identification of the spin adduct (Zielonka et al., 2016a). Detection of ONOO^−^-specific Luc-Bz-NO_2_, in combination with bioluminescence-imaging, was proposed for identification of this oxidant and its formation in activated macrophages has been demonstrated (Zielonka et al., 2016a).

Hypochlorite is another oxidant that is able to convert PCL-1 to Luc-OH (Sieracki et al., 2013). In a subsequent reaction with HOCl, Luc-OH produces 7′-chloroluciferin (Luc-OH-Cl, Scheme 7D) (Zielonka et al., 2016a). Therefore, Luc-OH-Cl detection in addition to bioluminescence *in vivo* may help identify HOCl as an oxidant responsible for the bioluminescence signal.

In 2013, the small-molecular-weight boronate pro-luciferin bioluminescent probe PCL-2 was proposed for dual-analyte *in vivo* imaging (Van De Bittner et al., 2013). This imaging system relies on *in situ* formation of luciferin in a condensation of 6-hydroxy-2-cyanobenzothiazole, which is uncaged by H_2_O_2_, and D-cysteine is produced upon the action of caspase 8 on pentapeptide. The disadvantage of this system for the sole purpose of detecting biological oxidants is the need to simultaneously unmask the two partners necessary to produce luciferin.

The described pro-luciferin bioluminescent probes were successfully applied for the bioluminescent detection of ONOO^−^ in cell culture studies (Sieracki et al., 2013; Zielonka et al., 2016a) and for the bioluminescent imaging of biological oxidants *in vivo* in mice (Van De Bittner et al., 2010, 2013; Cheng et al., 2020).

### Selectivity of Boronate-Based Probes

Meaningful application of the probes to detect a specific analyte requires knowledge of the probe's chemistry and, thus, selectivity. Over the last two decades, hundreds of promising new redox probes have been reported and their selectivity toward a single oxidizing species claimed. This is also true for boronate-based redox probes, which initially were claimed as selective for H_2_O_2_, while reports claiming their selectivity toward ONOO^−^ or HOCl were also published. Examples include the PF3 probe (**18**, also known as Fl-B, Table 1), which was proposed for selective detection of H_2_O_2_ (Dickinson et al., 2010) but later demonstrated to react with HOCl and ONOO^−^ and applied for monitoring ONOO^−^ in a cellular system (Rios et al., 2016). The PR1 probe (**8**, Table 1), proposed for the detection of H_2_O_2_ (Miller et al., 2005) and ONOO^−^ (Weber et al., 2018), represents an interesting case where initial oxidative deboronation of both boronate groups will result in the formation of dihydroresorufin, which in the presence of O_2_ will generate H_2_O_2_ (Maeda et al., 2000), driving further oxidation of the probe. In such a case, ONOO^−^-initiated probe oxidation may lead to H_2_O_2_ formation, and partial sensitivity of the fluorescence signal to catalase, an enzymatic H_2_O_2_ scavenger. In another example, the TCF-BOR2 probe (**43**, Table 1) was proposed for selective detection of HOCl (Shu et al., 2015), ONOO^−^ (Sedgwick et al., 2017), and H_2_O_2_ (Choudhury et al., 2019). As discussed, most boronate probes will respond to all three oxidants, as well as other oxidants listed above, at least in pure chemical systems. Clearly, many claims of the selectivity of boronate redox probes toward a single oxidizing species were due to the lack of expertise/appreciation of the limitations and potential pitfalls in studying the reactivity of several members of ROS.

Following are some typical pitfalls/limitations in studies of the probes' responses to different oxidants. It is our hope that this will help the community characterize new redox probes.

1. **Organic solvent**. Many cell-permeable probes are more soluble in organic solvents than in water. Therefore, their stock solutions are prepared in those solvents, typically DMSO or ethanol, due to their relatively low cytotoxicity. Those solvents may, however, scavenge the tested oxidant, resulting in false-negative. For example, the lack of response of the probe to the hydroxyl radical or HOCl may be due to scavenging of those species by DMSO. DMSO was shown to block oxidation of the CBA probe by HOCl (Zielonka et al., 2016a). When the use of organic solvents is necessary, their ability to compete for the oxidant of interest should be considered. One has to remember that even at 0.1% by vol., the organic solvent would equate to millimolar concentrations. For instance, 0.1% (by vol.) DMSO corresponds to a 14 mM concentration, three orders of magnitude higher than the typical concentration of the probe! When the use of an organic solvent cannot be avoided, to limit its interference and minimize the final concentration of the solvent, a high concentration stock solution (0.1 M or higher) of the probe should be made and serially diluted in the assay medium. In addition, the choice of solvent should be based both on its compatibility with cells and on its minimal reactivity toward the species to be detected. Always, the final concentration of the probe and solvent should be calculated and the ability of the probe to outcompete the solvent for the species of interest should be established. The ability of other solution components (e.g., buffer, metal chelator) to compete for the oxidants tested should be also considered.
2. **Excess of the oxidant over the probe**. Studies of the oxidation of boronates by H_2_O_2_ can be conveniently carried out using an excess of this oxidant. This is because, in most cases, the phenolic product is not reactive toward H_2_O_2_. This is not, however, the case for many other oxidants. As demonstrated for simple arylboronates, under the conditions of excess HOCl, the phenolic product undergoes chlorination, resulting in its consumption (Sikora et al., 2009). The same applies to many other oxidants, including HO^•^, ^•^NO_2_, and ${{\text{CO}}_{3}}^{•-}$, which are also the products of the decomposition of ONOO^−^ or ${{\text{ONOOCO}}_{2}}^{-}$. The use of an excess of the probe over the oxidant should help avoid the product consumption and better mimic the conditions in cell-based experiments where oxidants are very rarely present in excess of the probe.
3. **Using a bolus addition of an unstable oxidant**. In many cases, the oxidizing species to be studied is not stable and decomposes within the time of mixing. This applies not only to highly unstable free radicals but also, for example, to ONOO^−^. Efficient mixing of the probe with bolus ONOO^−^ requires good manual skills as well as some experience. Our typical protocol involves rapidly and vigorously mixing the solution of the probe placed in a 1.5 mL microcentrifuge tube with a small volume of ONOO^−^ solution placed inside the clean, dry tube cap. Another example is ${{\text{O}}_{2}}^{•-}$: Although it can be prepared as a stable solution of potassium superoxide (KO_2_) in an anhydrous DMSO, adding it to an aqueous buffer will result in rapid dismutation at the water-DMSO phase interface, the loss of ${{\text{O}}_{2}}^{•-}$, and the potential formation of not-yet-characterized DMSO-derived oxidants. Similarly, directly adding grains of KO_2_ powder to a buffered aqueous solution will result in efficient dismutation and loss of ${{\text{O}}_{2}}^{•-}$ at the KO_2_ solid/water interface. In the case of ${{\text{O}}_{2}}^{•-}$, ^•^NO, and ONOO^−^, chemical or biochemical sources can alternatively (or complementarily) be used to produce a slow, steady, and homogenous flux of the species and enable rigorous characterization of the probe's reactivity (Sikora et al., 2009; Zielonka et al., 2010).
4. **The stoichiometry of the reaction is higher than expected**. The determined stoichiometry of the reaction of mono-boronated probes with H_2_O_2_, HOCl, and ONOO^−^ is 1:1 (Zielonka et al., 2012a). It is expected that the stoichiometry may increase to 2:1 for a diboronated probe. A higher ratio of the oxidant to the probe may point to the loss of the oxidant by the interaction with a reaction mixture component, or a differential chemical reactivity. This may affect the probe's selectivity, and the reasons for the altered stoichiometry should be determined.
5. **Product formation is slower than the lifetime of the oxidant studied**. Direct reaction of the probe with the oxidant results in a decreased lifetime of the oxidant. Therefore, after the bolus addition, formation of the oxidation product should occur on a time scale shorter than the lifetime of the oxidant. Delayed formation of the product may occur when a self-immolative moiety is used in the probe design. In such case, the dynamics of elimination of the self-immolative moiety may control the kinetics of product formation. In other cases, delayed formation of the detectable product would bring into question the identity of the species responsible for probe oxidation. For example, ONOO^−^ decomposes within seconds. If the product formation is slower, it could indicate that ONOO^−^ is not the actual oxidant.

In addition to the points above, one must consider the effect of cellular milieu on the probe's reactivity. For example, in cells, the ability of boronate probes to intercept HOCl may be limited due to rapid HOCl reaction with thiols and amines, as discussed above. Conversely, strong one-electron oxidants, unreactive toward most boronate probes, may produce amino acid hydroperoxides, established oxidants of the boronate compounds. Clearly, knowledge of the chemical reactivity of boronates must be a pre-requisite of any studies of their fate and selectivity in cellular systems.

Although the rate constant of the reaction of boronate probes with different oxidants varies over several orders of magnitude, determining which oxidant is responsible for probe oxidation in a cell-based experiment may be not trivial, as all oxidants produce the same major product. Two complementary approaches have been proposed to identify the oxidants involved: (i) the use of species-specific enzymatic scavengers and inhibitors of the sources of the oxidants, and (ii) the profiling of the probe-derived products formed and identification of ONOO^−^- and HOCl-specific products (Zielonka et al., 2013a; Hardy et al., 2018). To prevent H_2_O_2_-mediated boronate oxidation, catalase may be used as an enzymatic scavenger, while inhibitors of the enzymatic sources of ${{\text{O}}_{2}}^{•-}$ and H_2_O_2_ may block H_2_O_2_ formation. To prevent ONOO^−^-mediated oxidation, superoxide dismutase (an enzymatic scavenger of ${{\text{O}}_{2}}^{•-}$) and inhibitors of nitric oxide synthases or enzymatic ${{\text{O}}_{2}}^{•-}$ sources may be used. In the case of HOCl-mediated oxidation, inhibitors of the MPO enzyme or of the enzymatic sources of ${{\text{O}}_{2}}^{•-}$ and H_2_O_2_ may be used to prevent probe oxidation. It should be noted that inhibitors of ${{\text{O}}_{2}}^{•-}$ sources are expected to inhibit the formation of all three mentioned oxidants, and thus may not be sufficient for identification purposes. However, they may help in establishing the source of the oxidant. Cell-permeable derivatives of antioxidant enzymes, or genetic manipulation of their cellular expression, may help in the modulation of intracellular oxidants. While using non-enzymatic, small-molecule scavengers would be a convenient route to identify the oxidizing species, the “inconvenient truth” is that, similar to redox probes, most small molecule chemical scavengers lack the sufficient selectivity to provide unequivocal assignment.

## Boronate-Based Redox Probes in the Studies of NADPH Oxidases

In addition to mitochondrial electron transport chain, NADPH oxidases (or Nox enzymes) are regarded as the major source of cellular ${{\text{O}}_{2}}^{•-}$ and H_2_O_2_. While an array of probes and assays have been developed over the last 30 years, in order to monitor Nox activity, most probes suffer from serious limitations, including the potential to generate ${{\text{O}}_{2}}^{•-}$ and H_2_O_2_ by the probes themselves (Zielonka et al., 2013b, 2017a; Kalyanaraman et al., 2016). Boronate redox probes have emerged as a new tool to study the activity and role of NADPH oxidases in cellular redox signaling and immune response (Dickinson et al., 2011a; Brewer et al., 2015; Zielonka et al., 2017a).

First application of boronate-based probes to study Nox-derived H_2_O_2_ was reported in the very first paper on the synthesis and use of monoboronated fluorogenic probes (Miller et al., 2007). Stimulation of the EGFR-mediated cellular signaling led to increased fluorescence signal from the PG1 probe, and the signal was inhibited by apocynin, used as a Nox inhibitor. As the ability of apocynin to inhibit NADPH oxidases is a subject of controversy (Heumüller et al., 2008), additional tests would be required to corroborate the involvement of Nox and determine the Nox isoform in EGFR-mediated H_2_O_2_ production.

The first identified Nox isoform, NADPH oxidase-2 (Nox2), is abundant in immune cells, including neutrophils and macrophages. With the use of CBA (**26**) and FlAmBE (**22**), boronate redox probes, it has been demonstrated that simultaneous activation of Nox2 and inducible nitric oxide synthase in macrophages results in production of ONOO^−^ (Zielonka et al., 2012b). These boronate probes have been used in parallel with the probes for ${{\text{O}}_{2}}^{•-}$ and ^•^NO, establishing a platform for “global profiling” of ROS and reactive nitrogen species in cellular systems (Zielonka et al., 2012b, 2013a). CBA and Fl-B (**18**) probes also have been used to determine the production of ONOO^−^ by activated macrophages in the presence of erythrocytes (Prolo et al., 2015). Because erythrocytes contain a large amount of hemoglobin, they serve as a sink/scavenger of ^•^NO, preventing it from reacting with Nox2-derived ${{\text{O}}_{2}}^{•-}$. Fl-B was also instrumental in demonstrating that *Trypanosoma cruzi* uses the Fe-superoxide dismutase enzyme to resist being killed by macrophages *via* removing phagosomal (Nox2-derived) ${{\text{O}}_{2}}^{•-}$ and limiting ONOO^−^ formation (Martinez et al., 2019).

Also, CBA proved to be the probe of choice to monitor H_2_O_2_ produced in activated neutrophils. Activation of Nox2 by a phorbol ester (PMA) in differentiated HL60 cells led to production of H_2_O_2_ as a product of ${{\text{O}}_{2}}^{•-}$ dismutation, and stimulation of CBA oxidation. This process was utilized in the design of a high-throughput screening campaign for the discovery of novel inhibitors of Nox2 (Zielonka et al., 2014, 2019). A combination of the CBA probe and hydroethidine was used for simultaneous monitoring of H_2_O_2_ and ${{\text{O}}_{2}}^{•-}$, released from activated neutrophils. The assay is based on rapid HPLC analyses (90 s per injection) of the cell media, and quantitation of COH and 2-hydroxyethidium as the products of H_2_O_2_ and ${{\text{O}}_{2}}^{•-}$. With such a setup, quasi-real-time monitoring of ${{\text{O}}_{2}}^{•-}$ and H_2_O_2_ was utilized to demonstrate the differences in the production of both species by different NADPH oxidase isoforms (Nox2, Nox4, and Nox5) (Zielonka et al., 2014). Following the design of the high-throughput screening workflow for Nox2 inhibitors, a medium-sized library of bioactive compounds (2029 compounds) was screened, and several candidates for Nox2 inhibitors have been identified (Zielonka et al., 2016b). With the use of the *o-*MitoPhB(OH)_2_ probe (**76**) and establishment of cyclo-*o-*MitoPh as a specific marker for ONOO^−^ (Scheme 6B), it was shown that the compounds capable of blocking Nox2 activity decrease ONOO^−^ production in activated macrophages.

## Concluding Remarks

Boronate probes represent a new class of redox probes with clear advantages over widely used probes based on reduced fluorescent dyes (e.g., DCFH, DHR). The reaction kinetics, stoichiometry, and mechanisms for many boronate probes have been established and reported, providing an opportunity for meaningful application of boronates in the area of redox biology research. Development of boronate probes for *in vivo* applications and determination of their pharmacokinetic behavior may yield novel tools for studying the role of biological oxidants in various pathological conditions, including cancer, neurodegeneration, and cardiovascular diseases.

## Author Contributions

All authors contributed to the review concept, design, and bibliographic research. AS, JZ, RM, RS-I, JP, and RP prepared the first version of the manuscript. AS, KD, and AA prepared the tables and AS, KD, KP, RP, and RM prepared the schemes and figures. AS, JZ, and BK critically reviewed and prepared the final version of the manuscript. All authors accepted the manuscript in its final form.

## Conflict of Interest

The authors declare that the research was conducted in the absence of any commercial or financial relationships that could be construed as a potential conflict of interest.
