# Supplementary material for: Boronate-Based Probes for Biological Oxidants: A Novel Class of Molecular Tools for Redox Biology
Source: Front Chem. 2020 Sep 25;8:580899. doi: 10.3389/fchem.2020.580899 (PMC7545953; doi:10.3389/fchem.2020.580899)
Supplement: Supplementary file 1 [file Table_1.docx]

Boronate-based probes for biological oxidants: A novel class of molecular tools for redox biology

**SUPPLEMENTARY MATERIALS**

Adam Sikora^1*^, Jacek Zielonka^2*^, Karolina Dębowska^1^, Radosław Michalski^1^, Renata Smulik-Izydorczyk^1^, Jakub Pięta^1^, Radosław Podsiadły^3^, Angelika Artelska^1^, Karolina Pierzchała^1^, Balaraman Kalyanaraman^2^

^1^Institute of Applied Radiation Chemistry, Faculty of Chemistry, Lodz University of Technology, 90-924 Lodz, Poland

^2^Department of Biophysics and Free Radical Research Center, Medical College of Wisconsin, Milwaukee, WI 53226, United States

^3^Institute of Polymer and Dye Technology, Faculty of Chemistry, Lodz University of Technology, 90-924 Lodz, Poland

*** Correspondence:**Adam Sikora
[adam.sikora@p.lodz.pl](mailto:adam.sikora@p.lodz.pl)

Jacek Zielonka
[jzielonk@mcw.edu](mailto:jzielonk@mcw.edu)

**Supplementary Table 1.**

MS/MS parameters for the mass spectrometric detection of MitoB (Cocheme et al., 2012) and
o-MitoPhB(OH)_2_ (Zielonka et al., 2015, 2016b) probes, and of their oxidation/nitration products.

| Probe | Analyte | Parent ion | Fragment ion |
| --- | --- | --- | --- |
| MitoB | MitoB | 397.1 | 183.0 |
|  | MitoB-*d_15_* (i.s.) | 412.2 | 191.1 |
|  | MitoP | 369.1 | 183.0 |
|  | MitoP-*d_15_* (i.s.) | 384.2 | 191.1 |
| *o*-MitoPhB(OH)_2_ | *o*-MitoPhB(OH)_2_ | 397.0 | 135.0 |
|  | *o*-MitoPhB(OH)_2_-*d_15_* (i.s.) | 412.2 | 117.1 |
|  | *o*-MitoPhOH | 369.0 | 107.1 |
|  | *o*-MitoPhOH-*d_15_* (i.s.) | 384.1 | 278.1 |
|  | *o*-MitoPhNO_2_ | 397.9 | 262.1 |
|  | *o*-MitoPhNO_2_-*d_15_* (i.s.) | 413.1 | 277.2 |
|  | *cyclo-o*-MitoPh | 351.1 | 183.1 |
|  | *o-*MitoPhCH_3_ (i.s.) | 367.0 | 105.1 |
| i.s. – internal standard | | | |
